# Systematic proteomic and small RNA profiling of extracellular vesicles from cattle infected with a naturally occurring buparvaquone-resistant strain of *Theileria annulata* and from uninfected controls

**DOI:** 10.1186/s13071-025-06834-8

**Published:** 2025-06-10

**Authors:** Yijun Chai, Jin Che, Jinming Wang, Guiquan Guan, Hong Yin

**Affiliations:** 1 State Key Laboratory for Animal Disease Control and Prevention, Key Laboratory of Veterinary Parasitology of Gansu Province, Lanzhou Veterinary Research Institute, Chinese Academy of Agricultural Sciences, Lanzhou, People’s Republic of China; 2 Heilongjiang Provincial Key Laboratory of Zoonosis, College of Veterinary Medicine, Northeast Agricultural University, Harbin, Heilongjiang People’s Republic of China; 3 Jiangsu Co-Innovation Center for the Prevention and Control of Important Animal Infectious Disease and Zoonosis, Yangzhou University, Yangzhou, People’s Republic of China

**Keywords:** *Theileria annulata*, Extracellular vesicles, Proteomics, Small RNA sequencing, Host–pathogen interaction

## Abstract

**Background:**

Extracellular vesicles (EVs) play a pivotal role in host-parasite interactions, particularly in facilitating parasite pathogenesis and immune modulation, and are crucial mediators of intercellular communication. *Theileria annulata*, an apicomplexan parasite, induces severe alterations in host cells, promoting uncontrolled proliferation, resistance to apoptosis, and immune evasion. Although EVs contribute to these processes, the proteins and small RNA cargo involved in *T. annulata* infection remain incompletely characterized. In particular, little is known about EV profiles in infections caused by drug-resistant field strains.

**Methods:**

In this study, we conducted systematic proteomic and small RNA profiling of EVs derived from naturally occurring buparvaquone-resistant *T. annulata* (Xinjiang Kashi strain) infected and uninfected bovine sera to investigate infection-induced alterations. Additionally, EVs were isolated from *T. annulata*-infected bovine immune cells to determine the protein and microRNA (miRNA) compositions of EVs secreted by specific immune cell types. Label-free liquid chromatography–tandem mass spectrometry proteomics and small RNA sequencing were employed to identify EV-associated proteins and miRNAs, followed by functional enrichment analysis to explore key host-parasite regulatory pathways.

**Results:**

Our analysis identified 2580 proteins and 6635 miRNAs in EVs derived from *T. annulata*-infected bovine serum and immune cell types, many of which are implicated in parasite development, host invasion, and immune modulation. Significant alterations were observed in the EV cargo from infected sera, including enrichment of vesicular proteins and miRNAs associated with immune regulation, metabolic reprogramming, and host–pathogen interactions. Furthermore, functional enrichment analyses highlighted key pathways such as ECM-receptor interactions, oxidative phosphorylation, and proton transport, underscoring the role of EVs in host immune modulation. Supplementary analysis of EVs from infected immune cells provided further insights into the cell type-specific contributions.

**Conclusions:**

This study comprehensively characterized the infection-induced changes in serum-derived EVs associated with a naturally occurring buparvaquone-resistant *T. annulata* infection. It offers novel insights into how *T. annulata* exploits EVs to manipulate host responses. The identification of unique EV-associated proteins and miRNAs highlights their potential as biomarkers and therapeutic targets for *Theileria* infections. These findings contribute to a deeper understanding of host-parasite interactions and lay the foundation for future investigations into EV-mediated pathogenesis and immune evasion.

**Graphical abstract:**

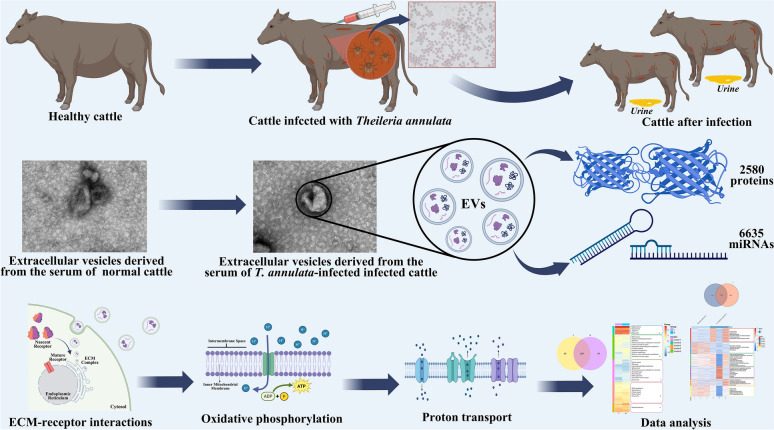

**Supplementary Information:**

The online version contains supplementary material available at 10.1186/s13071-025-06834-8.

## Background

*Theileria annulata* is a tick-borne apicomplexan parasite responsible for tropical theileriosis, a disease that threatens approximately 250 million cattle across Southern Europe, North Africa, and Asia, and causes substantial economic losses, particularly in developing countries [1, 2]. The life cycle of the parasite includes sexual and asexual stages, which occur in tick vectors and mammalian hosts, respectively [2, 3]. Upon transmission through tick bites, infective sporozoites invade bovine nucleated blood cells, preferentially targeting macrophages and B cells [4]. Schizogony is then initiated, leading to rapid intracellular multiplication and production of schizonts [5]. Infected leukocytes exhibit cancer-like traits, including unchecked proliferation and excessive cytokine production, which significantly contribute to disease pathogenesis [6]. Currently, an attenuated schizont cell line vaccine is used to control tropical theileriosis in Israel, Tunisia, Sudan, Egypt, India, Iran, Turkey, and China [7–10]; however, the underlying mechanism remains unclear. Furthermore, treatment relies heavily on buparvaquone, which raises concerns about emerging drug resistance [11]. These challenges highlight the necessity for novel vaccines and alternative therapeutic targets.

Extracellular vesicles (EVs) have emerged as essential mediators of intercellular communication, facilitating the transfer of biomolecules, such as proteins, lipids, and RNAs to recipient cells, thereby influencing cellular signaling, immune responses, and pathogen-host interactions [12, 13]. EVs, including exosomes and microvesicles, are involved in various biological processes, such as immune modulation, gene regulation, and cellular homeostasis, largely through non-coding RNAs, such as microRNAs (miRNAs), long non-coding RNAs, and circular RNAs [14, 15]. It is hypothesized that parasites such as *T. annulata* exploit EVs to modulate host–pathogen interactions by transferring parasite-derived molecules, including proteins and miRNAs, into host cells. Notably, miRNAs such as miR-155, miR-126-5p, and miR-34c-3p, which are upregulated during *T. annulata* infection, regulate key pathways, including the c-Jun N-terminal kinase (JNK)/c-Jun and protein kinase A pathways. These regulatory mechanisms contribute to host cell transformation, enhanced parasite survival, and the persistence of infection [13, 16–19].

Many parasite-secreted proteins are encapsulated within EVs that deliver their molecular cargo to host cells through membrane fusion, receptor-mediated uptake, and phagocytosis [20, 21]. EV-associated proteins play essential roles in parasite biology by influencing adaptation, differentiation, and survival throughout the parasite’s life cycle. In parasitic diseases caused by *Schistosoma japonicum*, *Leishmania* spp., *Plasmodium* spp., and *Toxoplasma gondii*, EVs transport key molecular components that regulate parasite development and host interactions [22–25]. However, while *T. annulata*-derived EVs have been partially characterized at the protein level, their combined proteomic and small RNA contents, particularly in the context of host–pathogen interactions, remain largely unexplored [26].

To address this gap, we conducted systematic proteomic and small RNA analyses of EVs derived from *T. annulata*-infected and *T. annulata*-uninfected bovine sera to identify infection-induced changes in EV cargo. Additionally, EVs from *T. annulata*-infected bovine immune cells were analyzed to obtain supplementary insights into cell type-specific EV compositions (detailed in the Additional files). By characterizing both the protein and miRNA components of these EVs, this study aimed to enhance our understanding of *T. annulata*-mediated molecular regulation and identify potential targets for vaccine and therapeutic development.

## Methods

### EV isolation from cattle sera

Serum samples were collected from cattle experimentally infected with *T. annulata* (Xinjiang Kashi strain; TaXJS) at 3–4 weeks post-infection, and samples from uninfected cattle within the same herd and of the same age range were used as controls. The experimental infection was conducted via intravenous injection of bovine blood containing merozoites of the TaXJS strain, which had been cryopreserved in the Vectors and Vector-borne Diseases Laboratory at the Lanzhou Veterinary Research Institute (Lanzhou, Gansu). The *T. annulata* (TaXJS) parasites were obtained from the same laboratory. This strain has been previously characterized as naturally occurring buparvaquone-resistant, as confirmed by drug sensitivity assays and* TaPIN1* gene mutation analysis [27–30].

EVs were isolated from 4 mL of serum, using the exoEasy Maxi Kit (QIAGEN, Germany) based on a membrane affinity spin column method, following the manufacturer’s guidelines. Purified EVs were resuspended in phosphate-buffered saline and stored at − 80 °C for subsequent analyses.

### EV isolation from cell culture

EVs were also isolated from *T. annulata* (Xinjiang Kashi strain, TaXJS)-infected bovine immune cells (lymphocytes, B cells, and dendritic cells) to gain additional insights into cell type-specific EV compositions [31, 32]. The TaXJS strain was originally isolated from naturally infected cattle and is characterized as a naturally occurring buparvaquone-resistant strain [27, 29, 30]. The isolation procedure followed standard ultracentrifugation protocols as previously described [33]. A detailed description of this procedure is provided in the Supplementary methods (Additional file 1).

### EV characterization

#### Transmission electron microscope analysis

EV morphology was assessed using a transmission electron microscope. Briefly, a 10-μL aliquot of each EV suspension was placed onto 200-mesh Formvar/carbon-coated copper grids (Agar Scientific, UK) for 10-min absorption. Excess fluid was removed, and the grids were negatively stained with 3% phosphotungstic acid (pH 7.0) for 1 min at room temperature. After air-drying, the grids were visualized using a Hitachi HT7800 transmission electron microscope (Hitachi, Japan) at 80 kV.

#### Nanoparticle tracking analysis

EV size distribution and concentration were determined using a NanoSight N30E system (Nanosight, Wiltshire, UK) [34]. Each sample was analyzed in triplicate, and the results were processed using NF Profession software (version 1.17, China) [35].

#### Label-free quantitative proteomic analysis

Proteins from EVs were extracted using a standard lysis protocol with urea buffer (8 M urea, 100 mM Tris–HCl, pH 8.0), followed by reduction with 5 mM dithiothreitol at 56 °C for 30 min and alkylation with 10 mM iodoacetamide at room temperature for 30 min in the dark. The protein concentration was determined using a bicinchoninic acid assay. For digestion, proteins were diluted with 50 mM ammonium bicarbonate to a final concentration of 1 M urea, followed by digestion with sequencing-grade modified trypsin (Promega, USA) at a 1:50 enzyme-to-substrate ratio overnight at 37 °C.

Peptides were desalted using C18 spin columns (Thermo Fisher Scientific, USA) and dried under vacuum. The samples were reconstituted in 0.1% formic acid and analyzed using an Ultimate 3000 High-performance liquid chromatography system (Thermo Fisher Scientific) coupled to a Q Exactive HF-X mass spectrometer (Thermo Fisher Scientific). Peptides were separated on an EASY Spray C18 column (75 μm × 25 cm) with a gradient of 4–35% acetonitrile in 0.1% formic acid over 90 min at a flow rate of 300 nL/min.

The mass spectrometer was operated in the data-dependent acquisition mode with a full scan range of 300–1800 m/z at a resolution of 70,000. The tandem mass spectrometry spectra were acquired at a resolution of 17,500 with a dynamic exclusion of 30 s. Raw data were processed using MaxQuant version 1.6 for protein identification and label-free quantification. Proteins were searched against the UniProt *Bos taurus* and *T. annulata* databases, allowing for a maximum of two missed cleavages and setting the carbamidomethylation of cysteine as a fixed modification and the oxidation of methionine as a variable modification. Quantitative data were normalized using the MaxLFQ algorithm in MaxQuant. Differentially expressed proteins (DEPs) were identified based on |log_2_ fold change|> 1 and *p* < 0.05. Functional enrichment analyses, including Gene Ontology (GO) and Kyoto Encyclopedia of Genes and Genomes (KEGG) pathway analyses, were performed to determine the biological relevance of the DEPs.

### Small RNA analysis and bioinformatics

Total RNA was prepared from triplicate samples using Trizol reagent (Thermo Fisher Scientific) according to the manufacturer’s guidelines. Small RNA libraries were prepared using the NEBNext^®^ Multiplex Small RNA Library Prep Kit (NEB, USA) according to the manufacturer’s protocol. Libraries were sequenced on a NovaSeq 6000 platform (Illumina, San Diego, CA) to generate 50-bp single-end reads. After sequencing, the raw reads were processed to remove low-quality reads, adapter sequences, and technical artifacts, such as adapter dimers and short or low-complexity reads, following established protocols [36]. Clean reads were aligned to the *B. taurus* and *T. annulata* genomes using BowTie software. Known miRNAs were identified by comparing the reads to miRBase v22 and novel miRNAs were predicted using mirDeep2. The identified miRNAs were used for subsequent bioinformatics analyses, as described below.

### Bioinformatics analysis

Functional enrichment analysis was performed on both proteomic and small RNA datasets to explore the biological significance of the DEPs and miRNAs. Raw data were analyzed using MaxQuant, with the National Center for Biotechnology Information *T. annulata* protein database (7627 proteins) as a reference. DEPs were identified based on the following criteria: *P*-value < 0.05 and |log_2_ fold change|> 1.

For small RNA sequencing data, differential expression analysis of miRNAs was performed using DESeq2, with differentially expressed miRNAs defined as those with a |log_2_ fold change| > 1 and *P*-value < 0.05. MiRNAs identified as potentially unreliable based on prior literature or internal validation were excluded from further analysis [37]. Target genes of differentially expressed miRNAs were identified by integrating predictions from the TargetFinder and miRanda databases (http://www.bioinformatics.com.cn/l) to enhance candidate reliability [38, 39].

GO and InterPro functional analyses were conducted using the InterProScan program to annotate proteins against a non-redundant protein database [40]. Additionally, the Clusters of Orthologous Groups and KEGG databases were used to analyze protein families and pathways and to determine the biological and functional properties of the identified proteins and miRNAs. DEPs and miRNAs were subjected to volcano plot analysis, cluster heatmap visualization, and enrichment analysis using GO, InterPro, and KEGG [40]. Cytoscape and the igraph package in R software (version 3.6.1) were used to construct and visualize interwoven networks of proteins and non-coding RNAs involved in various signaling pathways. These analyses provide insights into the molecular mechanisms by which *T. annulata* manipulates host cells through EV cargo [41].

## Results

### Identification of serum-derived EVs in *T. annulata*-infected and uninfected cattle

**Fig. 1A, B:**
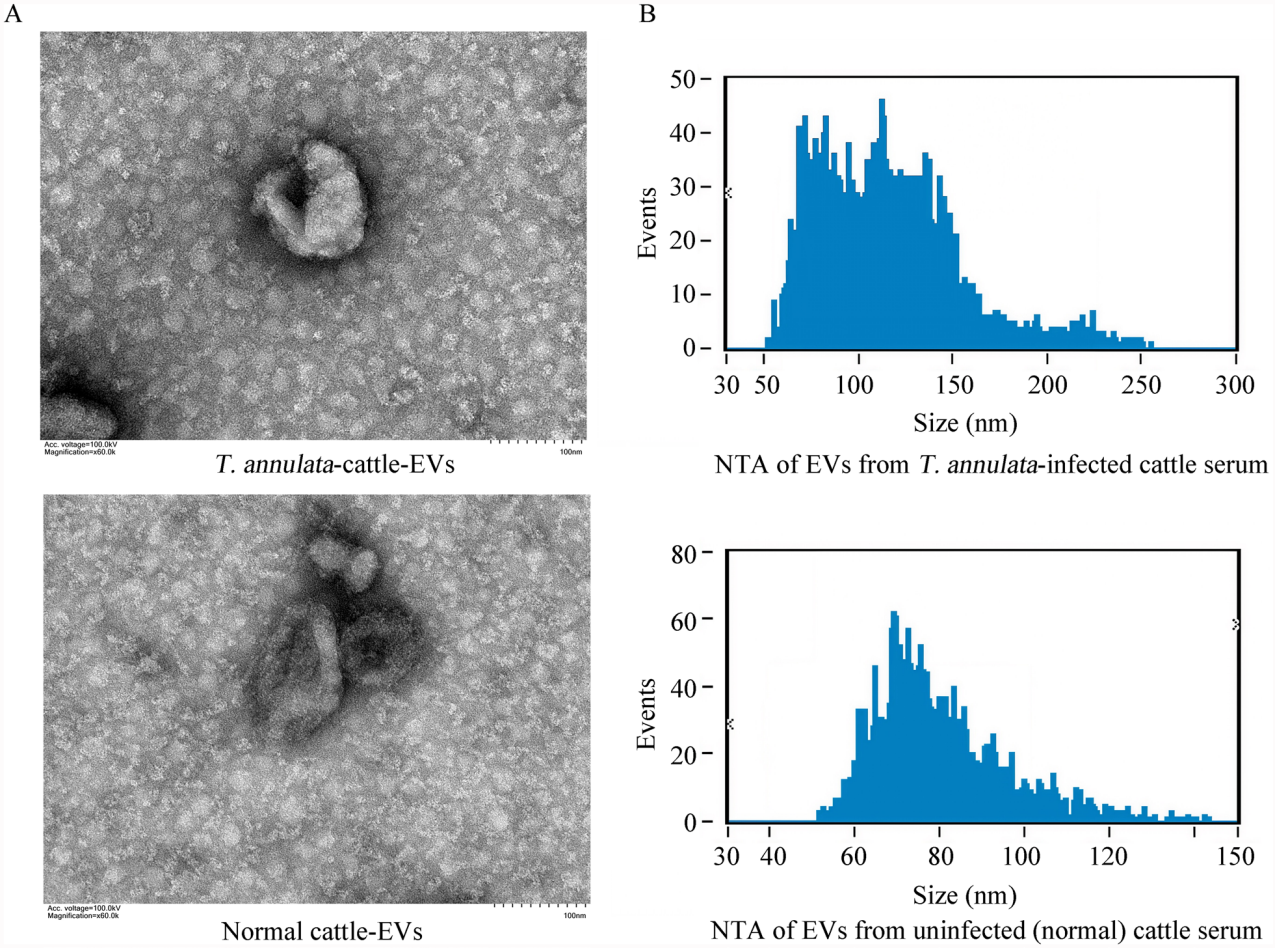
Quality analysis of isolated extracellular vesicles (*EVs*). **A** Representative transmission electron micrograph of EVs derived from the serum of *Theileria annulata*-infected and non-*T. annulata*-infected cattle. **B** Nanoparticle tracking analysis (*NTA*) describing the size and concentration of isolated EVs from the serum

The morphology, particle size, and concentration of the serum-derived EVs were systematically characterized to confirm their identity and uniformity. Transmission electron microscopy revealed that serum-derived EVs exhibited a typical cup-shaped morphology, with a size distribution ranging from 40 to 150 nm and an average diameter of approximately 100 nm, consistent with the expected characteristics of EVs (Fig. 1A). Nanoparticle tracking analysis demonstrated that EV concentrations differed significantly between the sera of infected and uninfected cattle, with infected cattle exhibiting a notable increase in EV production (Fig. 1B).

### EVs from *T. annulata*-infected bovine cell lines

Having identified infection-specific EVs in the serum of infected cattle, we aimed to determine which *T. annulata*-transformed cell types were the likely sources of these EVs. Therefore, we profiled the EVs from B cells (TaBC), macrophages (TaXJS), and dendritic cells (TaDC) (Additional file 1: Supplementary methods). Characterization confirmed typical EV morphology, size distribution (40–150 nm), and surface marker expression (Additional file 2: Fig. S1A–C). Proteomic analysis identified 15 T*. annulata*-derived proteins within these EVs (Additional file 3: Table S1), with significant variations observed in their proteomic profiles (Additional file 2: Fig. S2). Pairwise comparisons revealed distinct pathway enrichment patterns among the three cell lines, highlighting key differences in immune modulation, metabolic regulation, and signaling pathways (Additional file 2: Figs. S3, S4). We also performed principal component analysis to assess the reproducibility of miRNA expression levels across samples (Additional file 2: Fig. S5). MiRNA sequencing revealed both unique and shared expression patterns across the three EV types, with specific miRNAs demonstrating distinct regulatory roles (Additional file 2: Figs. S6, S7, S8). To provide a more comprehensive analysis, we compared protein expression across all five EV types using heatmap visualization (Additional file 2: Fig. S9) and assessed the proportion of small RNA species (Additional file 2: Fig. S10). A comparison of EV content between serum-derived and infected cell line-derived EVs revealed a substantial overlap in infection-associated proteins. Among the 19 infection-specific proteins identified in serum-derived EVs (Table 1), 16 were also detected in at least one of the three infected cell line-derived EVs (TaXJS, TaBC, or TaDC), including parasite-derived proteins such as actin 1, CD8^+^ T cell antigen Ta9, and HSP70 (Additional file 3: Table S1). However, three proteins (NP_001035046.1, XP_954369, and XP_954691) were not detected in any of the cell line-derived EVs, suggesting in vivo-specific packaging. Conversely, proteins such as glyceraldehyde-3-phosphate dehydrogenase and elongation factor 1 alpha were uniquely found in cell line-derived EVs, supporting the presence of cell type-specific EV compositions. These additional analyses enriched our dataset and provided deeper insights into the molecular landscape of *T. annulata*-associated EVs. Detailed EV characterization, proteomic, and miRNA analyses of these infected cell lines are made available in Additional file 4: Supplementary data.

**Table 1:** Serum extracellular vesicle (EV) proteins identified by liquid chromatography–tandem mass spectrometry before and after *Theileria annulata* infection

Protein^a^	Description	Gene identifier^b^	Non-infected^c^	Infected^d^
NP_001014904.1	Charged multivesicular body protein 1b	512033	None	Yes
NP_001029610.1	40S ribosomal protein S20	513222	None	Yes
XP_024848975.1	Annexin A3 isoform X1	518050	None	Yes
NP_001030494.1	THO complex subunit 4	537706	None	Yes
NP_001035046.1	Integrin alpha-M precursor	407124	None	Yes
NP_001039947.1	Sialic acid synthase	540602	None	Yes
NP_001068983.2	7-Methylguanosine phosphate-specific 5-nucleotidase	511449	None	Yes
XP_024855687.1	S-formylglutathione hydrolase isoform X2	535653	None	Yes
NP_001076965.1	Lactoylglutathione lyase	540335	None	Yes
XP_024841602.1	Gamma-tubulin complex component 2 isoform X1	781376	None	Yes
NP_001106714.1	Coronin-1B	510128	None	Yes
NP_001106792.1	Acyl-CoA-binding protein	768330	None	Yes
XP_024839428.1	Peptidyl-prolyl cis–trans isomerase FKBP5 isoform X3	535704	None	Yes
XP_024848875.1	Alpha-adducin isoform X11	507193	None	Yes
NP_786992.1	ADP-ribosyl cyclase/cyclic ADP-ribose hydrolase 1	327677	None	Yes
XP_002694568.2	EIF-2-alpha kinase activator GCN1 isoform X1	536143	None	Yes
XP_005207929.1	Ubiquitin carboxyl-terminal hydrolase isozyme L1 isoform X1	514394	None	Yes
XP_010817313.1	E3 ubiquitin-protein ligase NEDD4-like isoform X11	510003	None	Yes
XP_015315396.1	BOLA class I histocompatibility antigen, alpha chain BL3-6	507917	None	Yes
XP_951820	Uncharacterized protein	TA15710	None	Yes
XP_951821	Uncharacterized protein	TA15705	None	Yes
XP_954237	Uncharacterized protein	TA20390	None	Yes
XP_954369	Uncharacterized protein	TA21045	None	Yes
XP_954691	ABC transporter protein	TA19175	None	Yes

This table includes bovine proteins that were completely absent from all three replicates of the pre-infection samples, but consistently detected after *T. annulata* infection, indicating strong infection-associated EV markers

^a^Protein accession numbers were sourced from the National Center for Biotechnology Information Reference Sequence Database

^b^Gene identifiers correspond to entries in the National Center for Biotechnology Information Gene Database

^c^Absent from serum EVs

^d^Present in serum EVs

### Pairwise comparison of EVs proteomics

**Fig. 2A–D:**
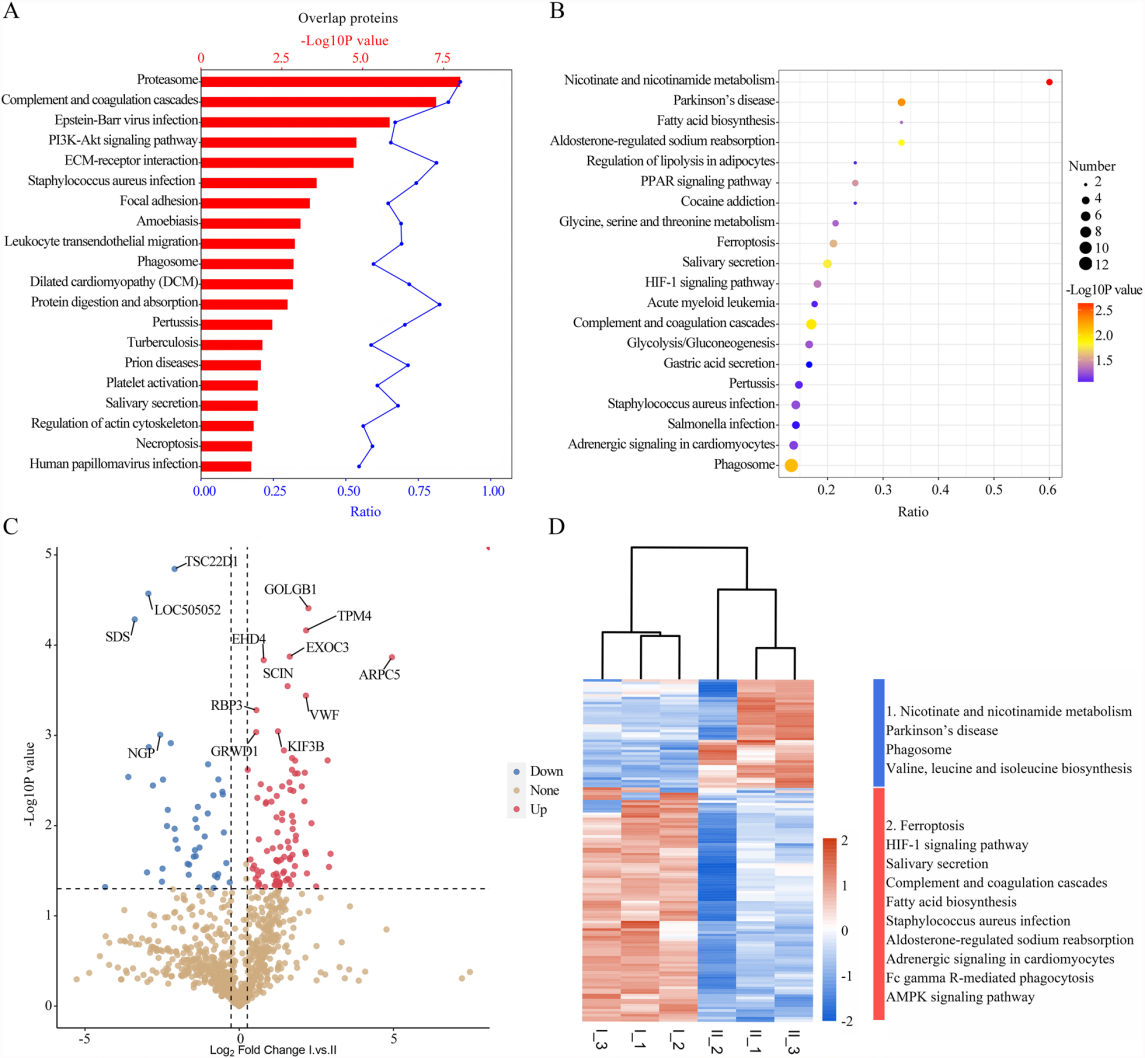
Comparative bioinformatics analysis of shared proteins and their regulatory potential across EVs. **A** Serum EVs from *Theileria annulata*-infected cattle versus healthy cattle. The* red bars* indicate -log_10_ (*P*-value), while the* blue dots* represent the proportion of candidate genes within the total pool of pathway-associated genes. **B** Kyoto Encyclopedia of Genes and Genomes (KEGG) bubble plots of overlapping proteins between serum EVs from *T. annulata*-infected versus healthy cattle. **C** Volcano plots displaying differentially expressed proteins (*P* < 0.05, |log_2_FC|≥ 1) for serum EVs from *T. annulata*-infected versus healthy cattle. Gene Ontology (GO) and KEGG analyses of upregulated protein clusters are shown, ranked by statistical significance. **D** Heatmaps depicting the expression levels of shared proteins between serum EVs from *T. annulata*-infected versus healthy cattle. The most significantly enriched pathways are indicated to the right of each heatmap. This annotation applies only to heatmaps; I and II denote normal bovine serum EVs and serum EVs from *T. annulata*-infected cattle, respectively

To investigate shared protein-coding genes across different EV groups, we performed heatmap analysis, volcano plots, and gene set enrichment analysis to identify DEPs (*p* < 0.05, |log₂FC|≥ 1; Fig. 2). Enrichment analysis of overlapping proteins (Fig. 2A) revealed that the most significantly enriched pathways in serum-derived EVs from *T. annulata*-infected cattle included protein proteasome-mediated protein processing, complement and coagulation cascades, PI3K-Akt signaling, ECM-receptor interaction, focal adhesion, leukocyte transendothelial migration, phagosome formation, protein digestion and absorption, platelet activation, regulation of the actin cytoskeleton, and necroptosis. The bubble plots (Fig. 2B) show that pathways such as nicotinate and nicotinamide metabolism, phagosome formation, and amino acid (valine, leucine, and isoleucine) biosynthesis were highly enriched in *T. annulata*-infected serum-derived EVs. Conversely, several pathways were downregulated, including ferroptosis, HIF-1 signaling, complement and coagulation cascades, fatty acid biosynthesis, Fc gamma R-mediated phagocytosis, and the AMP-activated protein kinase (AMPK) signaling pathway. Volcano plot analysis (Fig. 2C) further demonstrated significant protein expression changes between infected and control groups: 93 proteins were significantly upregulated and 43 significantly downregulated in serum-derived EVs from *T. annulata*-infected cattle (*p* < 0.05, |log₂FC|≥ 1). Heatmap clustering analysis (Fig. 2D) illustrated a clear distinction in biological pathway enrichment between *T. annulata*-infected cattle and uninfected controls, emphasizing the differential regulation of immune-related and metabolic pathways. Among the proteins identified in serum-derived EVs from *T. annulata*-infected cattle were enriched proteins related to nicotinate and nicotinamide metabolism, phagosome formation, and valine, leucine, and isoleucine biosynthesis. Five *T. annulata*-specific proteins were identified in the serum-derived EVs from infected cattle using liquid chromatography–tandem mass spectrometry (Table 1). Additionally, 19 bovine host proteins specifically associated with *T. annulata* infection were identified in the serum EVs from infected cattle (Table 1). In contrast, 15 T*. annulata*-derived proteins were detected in the EVs isolated from the three infected cell lines (TaXJS-EVs, TaBC-EVs, and TaDC-EVs), as listed in Table S1 (Additional file 3: Table S1). Notably, 16 of 19 host-derived proteins in serum-derived EVs were also detected in at least one of the three infected bovine cell line-derived EV samples (TaXJS, TaBC, or TaDC). The remaining three proteins NP_001035046.1 (integrin alpha-M precursor), XP_954369, and XP_954691 were not detected in any of the cell line-derived EVs (Additional file 3: Table S1).

### Bioinformatics of overlapping proteomes

**Fig. 3A–C:**
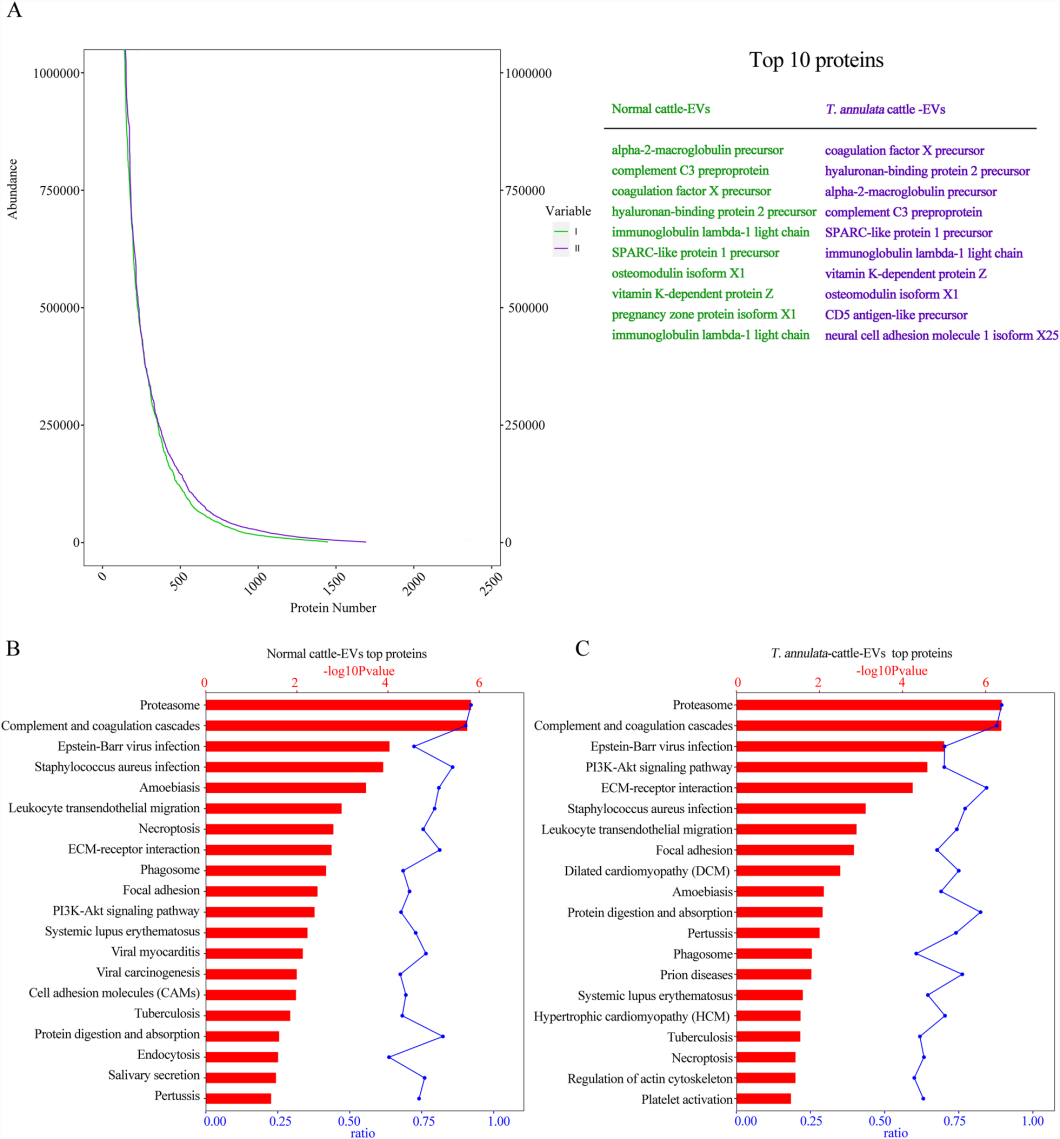
Network regulation involving top-loaded proteins in EVs. **A** Expression abundance curves for the serum EVs from *Theileria annulata*-infected and uninfected cattle, alongside the top 10 loaded proteins identified in each type. **B**, **C** KEGG pathway analysis of the top expressed proteins in **B** serum EVs from healthy cattle and **C** serum EVs from *T. annulata*-infected cattle. Pathways are ranked by -log_10_ (*P*-value). The* red bars* represent -log_10_ (*P*-value), and the* blue dots* indicate the proportion of candidate genes within the total pathway-related gene pool. For abbreviations, see Figs. 1 and  2

**Fig. 4A–E:**
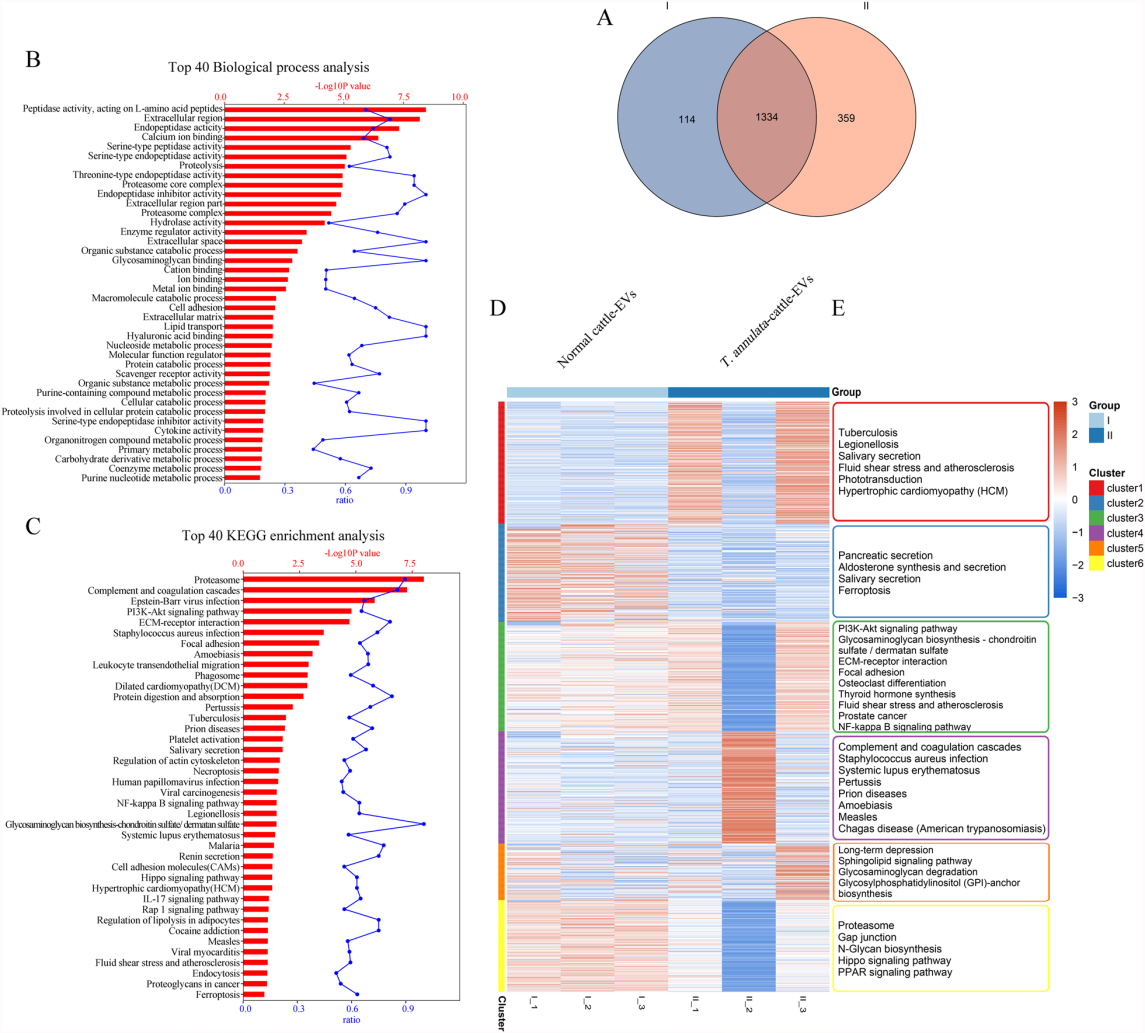
The overlapping proteins of normal bovine serum EVs and bovine serum EVs infected with *Theileria annulata* samples were involved in complex biological regulation. **A** Venn diagram of the shared proteins in normal cattle EVs (I) and serum of cattle with *T. annulata*-infected EVs (II). **B**, **C** GO and KEGG analyses of the shared proteins among the two EV types. The* red bars* represent the -log_10_ (*P*-values) and the* blue dots* are the proportion of candidate genes in the total pathway-related gene pool. **D** Heatmap of the levels of shared proteins among the two EV types. **E** The significantly enriched pathways. For abbreviations, see Figs. 1 and  2

We analyzed the expression abundance of serum-derived EVs from *T. annulata*-infected and uninfected cattle, and identified the top 10 EV-associated proteins (Fig. 3A). Both EV proteomes were involved in complex regulatory signaling networks, and the top 20 enriched pathways were ranked based on the values of -log_10_ (*P*-value; Fig. 3B, C). To evaluate the differences between the two EV proteomes, we constructed a Venn diagram to identify the unique and overlapping protein-coding gene clusters (Fig. 4). Bioinformatics analysis identified 1334 proteins in serum-derived EVs (Fig. 4A), regardless of infection status. These proteins were significantly involved in the pathways related to glycolysis, glycine, serine, and threonine metabolism, the peroxisome proliferator-activated receptor signaling pathway, fatty acid biosynthesis, and nicotinate and nicotinamide metabolism. GO analysis also demonstrated that these proteins were predominantly associated with EVs, membranes, protein-binding sites, and extracellular regions (Fig. 4B, C). Subsequently, we explored the shared proteomes of pre- and post-infection serum EVs and analyzed the associated protein-coding genes, using GO and KEGG enrichment to elucidate their biological roles (Fig. 4B, C). Certain KEGG pathways, such as HIV infection and schistosomiasis, appeared in the enrichment results owing to shared molecular interactions rather than direct biological relevance to *T. annulata* infection. Therefore, we focused on the pathways directly related to the host immune response, metabolic reprogramming, and vesicle-mediated communication.

Heatmap analysis revealed clear differences in the expression of shared proteins between EVs from uninfected and infected cattle serum (Fig. 4D, E). In cluster 1, proteins involved in tuberculosis, legionellosis, and other infection-related signaling pathways were enriched in infected serum EVs. Cluster 2 proteins showed reduced participation in aldosterone synthesis, secretion, and ferroptosis following infection. Clusters 3, 4, and 5 included proteins enriched both before and after infection with *T. annulata*. Cluster 6, which was mainly enriched in uninfected serum EVs, was associated with the proteasome, gap junction, N-glycan biosynthesis, hippo, and peroxisome proliferator-activated receptor signaling pathways.

### Identification of differentially expressed miRNAs in serum-derived EVs

**Fig. 5A–C:**
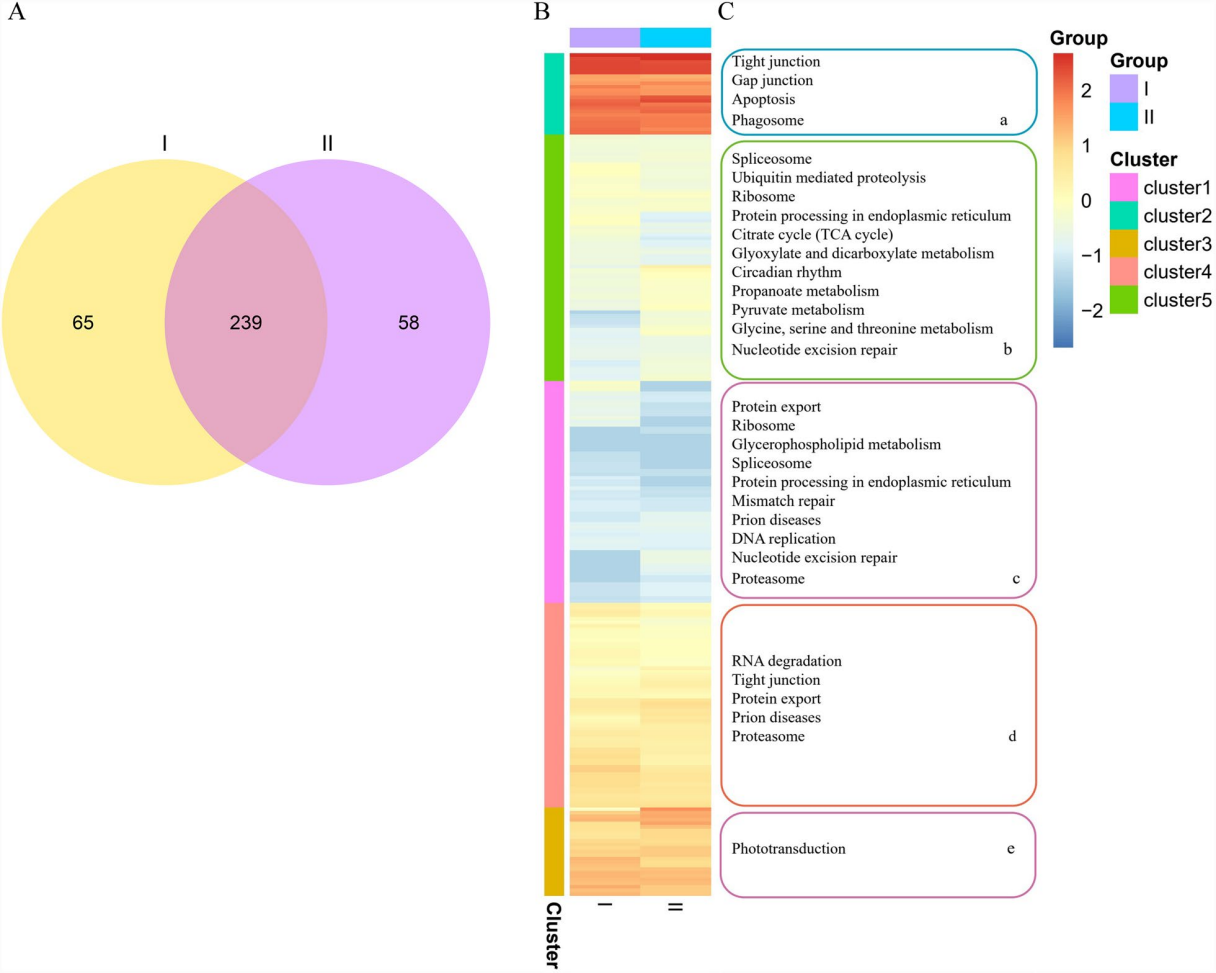
The signal regulation characteristics of specific microRNAs (miRNAs) derived from two EV samples. **A** Venn diagram of the miRNAs in normal bovine serum EVs and bovine-infected *Theileria annulata* serum EVs. **B** Heatmap of the shared miRNAs among the two EV types. **C** Significantly enriched pathways regulated by the different miRNA clusters in **B**. The* bubble size* represents the -log_10_ (*P*-value)

We next investigated the regulatory roles of miRNAs in serum-derived EVs from *T. annulata*-infected and uninfected cattle. A Venn diagram revealed 239 miRNAs shared between normal bovine serum EVs and bovine-infected *T. annulata* serum EVs (Fig. 5A). We analyzed their expression dynamics and potential involvement in the signaling pathway (Table 2). Additionally, 65 and 58 miRNAs were uniquely identified in uninfected and infected cattle serum EVs, respectively (Fig. 5A). The heatmap illustrates a shared miRNA distribution in EVs derived from bovine serum before and after infection (Fig. 5B). Two groups were represented: group I represented EVs from uninfected cattle, while group II represented EVs from infected cattle. Significant enrichment differences between the groups were observed across various clusters, each associated with distinct biological processes. Notably, clusters 1–5 showed marked enrichment of pathways, such as tight junctions, spliceosomes, ribosomes, and phototransduction (Fig. 5C). The most notable differences were observed in the pathways related to apoptosis, nucleotide excision repair, protein processing in the endoplasmic reticulum, and prion diseases. These differential enrichments suggest an infection-induced modulation of miRNA activity, likely contributing to alterations in cellular functions and host–pathogen interactions during *T. annulata* infection.

**Table 2:** Differentially expressed small RNAs (*sRNA*) in serum EVs before (*Pre*-) and after (*Post*-) *Theileria annulata* infection

sRNA	Pre-	Post-	Log_2_ fold change	*P*-value	Up/down^a^	Significant	Origin
bta-miR-143	38.35517	261.6527	2.798113	3.00E-06	Up	True	*Bos taurus*
bta-miR-206	5.336669	402.5107	6.356759	7.04E-23	Up	True	*B. taurus*
bta-miR-23a	44.58343	127.4908	1.510004	0.000571	Up	True	*B. taurus*
bta-miR-381	1.187444	18.62671	4.16326	8.46E-05	Up	True	*B. taurus*
bta-let-7d	80.24187	26.63947	− 1.6269	0.000576	Down	True	*B. taurus*
novel_210	8.107	0	− 5.06132	0.00164	Down	True	*T. annulata*
efu-let-7f	60.73957	26.76229	− 1.15172	0.020643	Down	True	*T. annulata*
ggo-let-7f	60.73957	26.76229	− 1.15172	0.020643	Down	True	*T. annulata*
hsa-let-7f-5p	60.73957	26.76229	− 1.15172	0.020643	Down	True	*T. annulata*
pma-let-7c	58.59428	26.04127	− 1.14033	0.021588	Down	True	*T. annulata*
rno-miR-3596d	60.73957	26.76229	− 1.15172	0.020643	Down	True	*T. annulata*
ssa-let-7f-5p	58.59428	26.04127	− 1.14033	0.021588	Down	True	*T. annulata*

sRNAs were mapped to *Bos taurus* and *T. annulata* genomes

^a^Indicates post-infection expression change

### Integrated analysis of EV proteins and small RNAs

Integrated proteomic and small RNA analyses of EVs derived from *T. annulata*-infected cells identified key molecules involved in immune regulation, metabolic reprogramming, and cell cycle processes. Proteins such as complement C3, ICAM-1, and annexin A6 were highly enriched across EVs from TaXJS, TaBC, and TaDC cells, whereas miRNAs, including bta-miR-148a, bta-miR-155, and bta-let-7i, were among the most highly expressed small RNAs. Proteins associated with metabolic processes, such as glyceraldehyde-3-phosphate dehydrogenase, are co-expressed with miRNAs such as bta-miR-206 and bta-let-7i, which are involved in regulating mitochondrial function and fatty acid metabolism. These molecules were consistently co-enriched in pathways related to ribosomal function, tight junctions, and complement system regulation, indicating their role in modulating host immune responses, protein synthesis, and metabolic activity during infection. Significant changes in both protein and miRNA expression were observed between pre- and post-infection EVs, with post-infection EVs showing the upregulation of pathways linked to immune evasion, oxidative phosphorylation, and apoptosis regulation. Protein–protein interaction network analysis further highlighted the involvement of both proteins and miRNAs in ECM-receptor interaction, cell cycle control, and AMPK signaling (Additional file 2: Fig. S4).

## Discussion

EVs, including exosomes and microvesicles, are lipid bilayer-enclosed structures secreted by various cell types and found in biological fluids. They serve as critical mediators of intercellular communication and participate in diverse physiological and pathological processes such as immune regulation, cellular signaling, and disease progression [42]. Growing evidence highlights the key role of EVs in host–pathogen interactions, particularly in parasitic infections. In this context, EVs facilitate processes such as immune suppression, metabolic reprogramming, and pathogen persistence [43, 44]. Consistent with findings from other pathogenic or disease-related models, EVs have been shown to encapsulate bioactive cargo, including small RNAs and lipids, which may mediate intercellular signaling and potentially contribute to disease progression [45, 46]. To enable a robust comparative analyses, we refined EV isolation and purification methods and characterized EVs derived from *T. annulata*-infected bovine cell lines (leukocytes, B cells, and dendritic cells). These EVs were compared with those isolated from both normal and infected bovine serum. Using ultracentrifugation combined with filtration-based approaches, we aimed to comprehensively define the molecular composition of EVs and evaluate their roles in immune evasion and metabolic reprogramming [47].

Proteomic analysis identified several highly conserved proteins, such as actin 1 and heat shock 70 kDa protein, in EVs isolated from *T. annulata*-infected bovine immune cell lines (TaXJS, TaBC, and TaDC; Supplementary Table S1). These proteins have been reported in host-derived EVs and parasite proteomes from other apicomplexan infections, including *Plasmodium falciparum*, *Toxoplasma gondii*, *Schistosoma*, and *Leishmania*, as well as in *Theileria annulata* schizont proteomic studies [48–50]. While these molecules are commonly associated with protein stabilization, stress responses, and EV biogenesis, their presence in EV preparations could also reflect a background of parasite lysis or cellular debris. Therefore, further validation is needed to determine whether these proteins are actively incorporated into EVs or are background contaminants. In contrast, other host proteins, such as glyceraldehyde-3-phosphate dehydrogenase and histone H4, were detected only in specific cell line-derived EVs, possibly indicating cell type-specific EV content or methodological differences. Other proteins, including 60S ribosomal protein L2/L8, elongation factor 1-alpha, and transitional endoplasmic reticulum ATPase, were absent from TaDC-derived EVs but were present in EVs derived from TaXJS and TaBC cell lines, suggesting potential differences in EV packaging related to immune cell subtype or parasite-host interaction context. Collectively, these findings emphasize the molecular complexity and potential selectivity of EV cargo from infected immune cells. In addition, one of the key parasite-derived proteins identified in our EV proteomic analysis was the CD8^+^ T cell antigen Ta9, which was detected in cell line-derived EVs but not in serum-derived EVs. Ta9 is a highly expressed schizont-stage protein containing multiple CD8^+^ T cell epitopes that has been validated as a potent immunogen capable of eliciting cytotoxic T lymphocyte responses in both naturally infected and vaccinated animals [51]. The presence of Ta9 in cell line-derived EVs in our study aligns with its known expression during the schizont stage, where it plays a critical role in host cell transformation. Functional studies have demonstrated that the C-terminal region of Ta9 activates the AP-1 transcription factor, a key regulator of *T. annulata-*induced leukocyte transformation. However, the presence of nuclear localization signals in this region remains a prediction based on sequence analysis [52]. Furthermore, the ectopic expression of Ta9 in bovine macrophages triggers a strong proinflammatory transcriptional response, which includes the upregulation of cytokines, chemokines, and proto-oncogenes, such as a kinase required for parasite-driven macrophage proliferation [53]. The identification of Ta9 in EVs from both serum infected during the merozoite stage and immune cells infected during the schizont stage not only highlights its role in host–pathogen interactions but also reinforces its potential as a vaccine candidate. Thus, our study provides new insights into the biological significance of Ta9, and suggests that this multifunctional protein, which is consistently detected across different developmental stages, may be selectively packaged into EVs and play important roles in both pathogenesis and protective immunity.

Our study provides the first comprehensive proteomic and miRNA profiling of EVs from *T. annulata*-infected cattle, with a primary focus on EVs derived from serum. Data on cell line-derived EVs are included as supplementary comparative data. By comparing serum-derived EVs from infected and uninfected cattle, we identified significant alterations in protein and miRNA expression levels, thereby elucidating the systemic impact of *T. annulata* infection. Unless otherwise noted, the discussion that follows centers on serum-derived EVs, which better represent systemic responses to *T. annulata* infection. Infected serum EVs were enriched in proteins involved in immune regulation, complement activation, and metabolic pathways. This observation highlights the critical role played by serum-derived EVs in host–pathogen interactions. Furthermore, significant differences in the host-derived miRNA cargo were observed, particularly in pathways related to apoptosis, phagocytosis, and ribosome function. Key host-derived proteins such as annexin A3, integrin alpha-M precursor, and ICAM-1 were specifically enriched in infected serum EVs, suggesting that their roles in modulating immune cell adhesion, extracellular matrix remodeling, and leukocyte trafficking processes are crucial to *T. annulata* pathogenesis [54–56]. The detection of acyl-CoA-binding protein further emphasizes the potential role of EVs in host metabolic adaptation, notably lipid transport, which is crucial for parasite survival.

Despite these notable advances, comparative analyses of EVs derived from various cell types infected with *T. annulata* are limited. Previous proteomic studies on EVs derived from bovine lymphosarcoma cells primarily utilized mass spectrometry and miRNA profiling, but lacked comprehensive functional characterization of EV cargo related to disease progression [26]. Earlier studies identified several host-derived proteins, including ICAM-1 and MMP-9, in EVs from *T. annulata*-infected cells, suggesting their roles in modulating host immune responses and cell migration [26]. However, previous research has predominantly focused on tumor suppressor-related miRNAs and overlooked other small RNAs and proteins that may be involved in immune modulation and metabolic adaptation. To understand the regulatory role of EV-associated miRNAs, we profiled their expression in serum EVs from infected cattle. Our study integrated label-free quantification proteomics with small RNA sequencing to provide a more comprehensive and detailed characterization of EV cargo. Proteomic and small RNA analyses were primarily performed using the host reference genome (*B. taurus*), and the majority of identified EV proteins and miRNAs were of host origin. Parasite-derived proteins were limited, and are listed in Table 1. Among the proteins uniquely identified in EVs from infected cattle sera (Table 1), five were annotated as *T. annulata*-derived, including both characterized (e.g., ABC transporter protein) and uncharacterized parasite proteins. These proteins were absent from all pre-infection replicates, but were consistently detected post-infection, supporting their infection-specific nature. Interestingly, parasite proteins (TA21045 and TA19175) were not found in EVs from any of the three infected bovine cell lines (Additional file 3: Table S1), suggesting that serum EVs may capture a broader range of parasite-secreted molecules, potentially originating from tissue-resident or circulating infected cells that are not represented in vitro. The presence of these proteins supports the hypothesis that parasite-derived cargo can be secreted into the circulatory system and may contribute to immune evasion, host cell modulation, or even serve as diagnostic biomarkers, such as the ABC transporter protein TA19175 and uncharacterized proteins (TA15710 and TA21045), which were exclusively detected in post-infection sera. However, further functional studies are required to elucidate the specific roles of these proteins. Bioinformatics analysis identified EV-associated proteins involved in regulating critical pathways, such as AMPK signaling, metabolic processes, and cell cycle regulation, thereby highlighting their significance in host-parasite interactions. Notably, several proteins, such as acyl-CoA-binding protein, annexin A3, E3 ubiquitin-protein ligase (NEDD4-like isoform X1), and ubiquitin carboxyl-terminal hydrolase isozyme L1 isoform X1, were identified in EVs post-*T. annulata* infection. The identification of such proteins suggests a potential role for parasite-derived EV cargo in modulating processes such as host lipid metabolism, energy homeostasis, and immune signaling.

Pathway enrichment analyses revealed the significant activation of focal adhesion, leukocyte transendothelial migration, and phagocytic pathways in infected serum EVs, supporting the hypothesis that *T. annulata* employs EVs to manipulate immune cell trafficking and tissue invasion. MicroRNA profiling further reinforced this concept, as we noticed a significant enrichment of miRNAs implicated in tumorigenesis, immune suppression, and metabolic regulation. These miRNAs included bta-miR-148a, bta-miR-155, and bta-let-7i, which are associated with critical signaling pathways including wingless-related integration site, transforming growth factor-beta, p53, AMPK, and mammalian target of rapamycin [57–59]. Hierarchical clustering analyses indicated significant expression changes in these miRNA clusters within the infected EVs, suggesting their involvement in infection-induced immune reprogramming [59]. Several EV-associated miRNAs identified in this study have been implicated in *Theileria*-induced host cell transformation and immune modulation. Notably, bta-miR-155, bta-miR-126-5p, and bta-miR-34c-3p were significantly upregulated in serum-derived EVs from infected cattle. miR-155 is involved in a positive feedback loop with c-Jun that stabilizes AP-1 activity, promoting leukocyte transformation in *T. annulata*-infected cells. It is also modulated in response to buparvaquone treatment [17]. miR-126-5p enhances the spread and virulence of infected macrophages by targeting JNK-interacting protein 2 and modulating JNK/c-Jun signaling [18]. miR-34c-3p, an infection-induced miRNA, shows a differential expression pattern between virulent and attenuated *T. annulata*-transformed macrophages and regulates protein kinase A activity independently of cAMP [19], pointing to a role in parasite-driven host cell reprogramming. While Zhao et al. [60] focused on the intracellular miRNA response to drug (buparvaquone) treatment, our study explored EV-associated miRNA profiles derived from the serum and cell culture supernatants of cattle infected with a drug (buparvaquone)-resistant strain (TaXJS), both in vitro and in vivo. Although we did not perform direct comparisons at the level of individual miRNAs, together, these two studies provide complementary insights: Zhao et al. [60] highlighted intracellular miRNA responses to chemotherapy, while our research uncovered extracellular miRNA signatures associated with infection by a drug-resistant strain. These differences emphasize the importance of integrating both cell- and EV-based miRNA profiles to better understand host-parasite interactions across diverse infection settings and parasite lineages.

Despite providing crucial novel insights, this study also has a few limitations. For example, the absence of uninfected control cell lines makes it difficult to directly link the observed EV changes to infection-specific effects. However, prior evidence showing distinct molecular profiles in EVs from attenuated vaccine strains versus virulent strains highlights the need for future comparative studies [18]. Additionally, functional validation using in vitro EV uptake assays and gene knockdown experiments is essential to confirm the biological significance of the identified EV cargo. Future studies employing single-cell transcriptomics and proteomics, both in EV-treated immune cells and in defined immune cell subsets isolated from *T. annulata*-infected animals, will be key to better understanding *T. annulata*-derived EV interactions with host immune cells in vivo, with a focus on specific immune cell subsets and their functional states. Furthermore, the identified EV protein and miRNA signatures hold potential as biomarkers for early disease diagnosis and severity assessment, as well as therapeutic targets for controlling tropical theileriosis.

Given their central role in *T. annulata* infection pathogenesis, EVs may offer a potential avenue for clinical application. Distinct protein and miRNA profiles in serum-derived EVs before and after *T. annulata* infection shed light on their utility as candidate biomarkers for early-stage diagnosis and disease monitoring (Tables 1, 2). Furthermore, immunomodulatory miRNAs and proteins in the EV cargo represent promising therapeutic targets. Insights into the differences in the composition of EVs between virulent and attenuated strains could facilitate vaccine development by identifying protective immune signatures. Future research should prioritize functional inhibition studies targeting EV-associated miRNAs and proteins to evaluate their diagnostic and therapeutic potential and to improve strategies for controlling tropical theileriosis.

## Conclusions

Proteomic and miRNA profiling of *T. annulata*-infected cattle EVs revealed distinct molecular signatures compared with uninfected controls. Infected serum EVs were enriched in immune-regulatory and metabolic pathway components, including those related to ECM-receptor interactions and oxidative phosphorylation. miRNA profiling identified key regulators involved in apoptosis and immune modulation. Importantly, cell-derived EVs exhibit unique infection-induced profiles that reflect parasite-driven modifications across different immune cell types. These findings suggest that EV-associated molecules could be helpful in the detection of infection and may lead to new treatments or vaccines.

## Supplementary Information

Additional file 1.

Additional file 2.

Additional file 3.

Additional file 4.

Additional file 5.

## Data Availability

The data supporting the findings of the study are available within the article and its supplementary materials.
