# Supplementary material for: Systematic proteomic and small RNA profiling of extracellular vesicles from cattle infected with a naturally occurring buparvaquone-resistant strain of Theileria annulata and from uninfected controls
Source: Parasit Vectors. 2025 Jun 10;18:221. doi: 10.1186/s13071-025-06834-8 (PMC12153157; doi:10.1186/s13071-025-06834-8)
Supplement: Supplementary file 1 — Additional file 1. [file 13071_2025_6834_MOESM1_ESM.docx]

**Additional file 1:**

**Supplementary Methods**

**Cell culture**

The *T. annulata*-infected bovine lymphocyte line (TaXJS), B cell line (TaBC), and dendritic cell line (TaDC) used in this study were all established from the *T. annulata* Kashi isolate from Xinjiang, which has been characterized as a naturally occurring buparvaquone-resistant strain based on drug sensitivity assays and TaPIN1 gene mutation analysis (cryopreserved at the Vectors and Vector-borne Diseases Laboratory of Lanzhou Veterinary Research Institute, LVRI, Lanzhou, China) [27-32]. All cell lines were cultured in RPMI 1640 medium (VivaCell, China) supplemented with 10% exosome-depleted fetal bovine serum (VivaCell, China) and maintained at 37°C in a humidified incubator containing 5% CO₂. Cells were passaged every 2-3 days to maintain log-phase growth.

**Extracellular Vesicle Isolation from Cell Culture Supernatants**

Extracellular vesicles from serum-free culture medium of cell culture supernatants were purified by serial centrifugation as previously described [33]. EVs were isolated from the serum-free culture supernatants of TaXJS, TaBC, and TaDC cell lines using a sequential centrifugation process, followed by ultracentrifugation. First, supernatants were subjected to differential centrifugation at 300g for 10 minutes, 2000g for 10 minutes, and 10,000g for 30 minutes at 4°C to remove cell debris and apoptotic bodies. The cleared supernatant was filtered through a 0.22μm membrane (Sigma, USA) and ultracentrifuged at 100,000g for 70 minutes using a Ti45 rotor (Beckman Coulter, USA). The EV pellet was resuspended in PBS, followed by a second ultracentrifugation at 100,000g for 70 minutes to eliminate contaminants. The final pellet was resuspended in PBS, and EV protein concentrations were determined using a Pierce BCA Protein Assay Kit (Thermo Fisher Scientific, USA). EVs were stored at -80°C for subsequent analyses.

**Transmission electron microscopy (TEM)**

EV morphology was assessed by transmission electron microscopy (TEM). Briefly, a 10μL aliquot of each EV suspension was placed onto 200-mesh formvar/carbon-coated copper grids (Agar Scientific, UK) and allowed to absorb for 10 minutes. Excess fluid was removed, and grids were negatively stained with 3% phosphotungstic acid (pH 7.0) for 1 minute at room temperature. After air drying, grids were visualized using a Hitachi HT7800 TEM (Hitachi, Japan) at 80 kV.

**Nanoparticle tracking analysis (NTA)**

The size distribution and number of EVs were analyzed by measuring the rate of Brownian motion of each particle using a NanoSight N30E instrument (Nanosight, Wiltshire, UK). The N30E can track the movement of individual particles in solution directly, thereby enabling the determination of particle size distribution as well as the number of nanoparticles [34]. The measurement procedure was performed as previously described [35]. Each sample was measured in triplicate and the software NF Profession (version 1.17, China) was used to capture and analyze the data.

**Western blotting for EV marker validation**

EVs (30μg of total protein) were lysed in RIPA buffer (Thermo Fisher Scientific, USA) containing protease inhibitors (Sigma-Aldrich, USA). Protein samples were separated by SDS-PAGE (4-20% Tris-glycine gels, Novex, USA) and transferred onto nitrocellulose membranes. Membranes were blocked in 5% skimmed dry milk and probed with primary antibodies against exosome markers TSG101 and HSP70 (Cell Signaling Technology, USA), followed by incubation with horseradish peroxidase (HRP)-conjugated secondary antibodies (Millipore, USA). Bands were visualized using an enhanced chemiluminescence kit (ECL, Thermo Fisher Scientific, USA).

**Label-free quantitative proteomic analysis (LFQ)**

Proteins from EVs were extracted using a standard lysis protocol with urea buffer (8 M urea, 100 mM Tris-HCl, pH 8.0), followed by reduction with 5 mM dithiothreitol (DTT) at 56°C for 30 minutes and alkylation with 10 mM iodoacetamide at room temperature for 30 minutes in the dark. Protein concentration was determined using a BCA assay. For digestion, proteins were diluted with 50 mM ammonium bicarbonate to a final concentration of 1 M urea, followed by digestion with sequencing-grade modified trypsin (Promega, USA) at a 1:50 enzyme-to-substrate ratio overnight at 37°C.

Peptides were desalted using C18 spin columns (Thermo Fisher Scientific, USA) and dried under vacuum. Samples were reconstituted in 0.1% formic acid and analyzed using an Ultimate 3000 HPLC system (Thermo Fisher Scientific, USA) coupled to a Q Exactive HF-X mass spectrometer (Thermo Fisher Scientific, USA). Peptides were separated on an EASY-Spray C18 column (75μm x 25 cm) with a gradient of 4% to 35% acetonitrile in 0.1% formic acid over 90 minutes at a flow rate of 300 nL/min.

The mass spectrometer was operated in data-dependent acquisition mode, with a full scan range of m/z 300-1800 at a resolution of 70,000. MS/MS spectra were acquired at a resolution of 17,500 with a dynamic exclusion of 30 seconds. Raw data were processed using MaxQuant software (version 1.6) for protein identification and label-free quantification. Proteins were searched against the UniProt *Bos taurus* and *T. annulata* databases, allowing for a maximum of two missed cleavages and setting carbamidomethylation of cysteines as a fixed modification and oxidation of methionines as a variable modification. Quantitative data were normalized using the MaxLFQ algorithm within MaxQuant. Differentially expressed proteins (DEPs) were identified based on a |log2 fold change| > 1 and P < 0.05. Functional enrichment analyses, including Gene Ontology (GO) and Kyoto Encyclopedia of Genes and Genomes (KEGG) pathway analysis, were performed to interpret the biological relevance of the DEPs.

**Small RNA analysis and bioinformatics**

Total RNA was prepared from triplicate samples using Trizol (Thermo Fisher) according to manufacturer’s guidelines. Small RNA libraries were prepared using the NEBNext® Multiplex Small RNA Library Prep Kit (NEB, USA) according to the manufacturer’s protocol. Libraries were sequenced on the Illumina NovaSeq 6000 platform, generating 50 bp single-end reads. After sequencing, the raw reads were processed to remove low-quality reads, adapters, and contaminants, following established protocols [36]. Clean reads were aligned to the *Bos taurus* and *T. annulata* genomes using Bowtie. Known miRNAs were identified by comparing the reads to miRBase v22, while novel miRNAs were predicted using mirDeep2. Differential expression analysis was performed using DESeq2, with differentially expressed miRNAs defined as those with a |log2 fold change| > 1 and p-value < 0.05. miRNAs identified as potentially unreliable based on prior literature reports or internal validation were excluded from further analysis [37]. Target genes of differentially expressed miRNAs were identified by integrating predictions from the TargetFinder and miRanda databases (<http://www.bioinformatics.com.cn/l>) to enhance candidate reliability [38,39].

**Bioinformatics analysis**

For both proteomic and small RNA datasets, functional enrichment analysis was performed to explore the biological significance of differentially expressed proteins (DEPs) and miRNAs. Raw data were analyzed using the MaxQuant software package, with the NCBI *T. annulata* protein database (7627 proteins) used as a reference. Differentially expressed proteins were identified based on a p-value < 0.05 and |log2 fold change| > 1. Gene Ontology (GO) and InterPro (IPR) functional analyses were conducted using the InterProScan program to annotate proteins against the non-redundant protein database [40]. Additionally, the Clusters of Orthologous Groups (COG) and Kyoto Encyclopedia of Genes and Genomes (KEGG) databases were used to analyze protein families and pathways, determining the biological and functional properties of the identified proteins and miRNAs. Differentially expressed proteins and miRNAs were subjected to volcano plot analysis, cluster heatmap visualization, and enrichment analysis of GO, IPR, and KEGG [31]. Cytoscape and the igraph package in R software (version 3.6.1) were employed to construct and visualize the interwoven networks of proteins and non-coding RNAs involved in various signaling pathways. These analyses provided insights into the molecular mechanisms by which *T. annulata* manipulates host cells through EV cargo [31].
