# Supplementary figures and images for: Systematic proteomic and small RNA profiling of extracellular vesicles from cattle infected with a naturally occurring buparvaquone-resistant strain of Theileria annulata and from uninfected controls
Source: Parasit Vectors. 2025 Jun 10;18:221. doi: 10.1186/s13071-025-06834-8 (PMC12153157; doi:10.1186/s13071-025-06834-8)

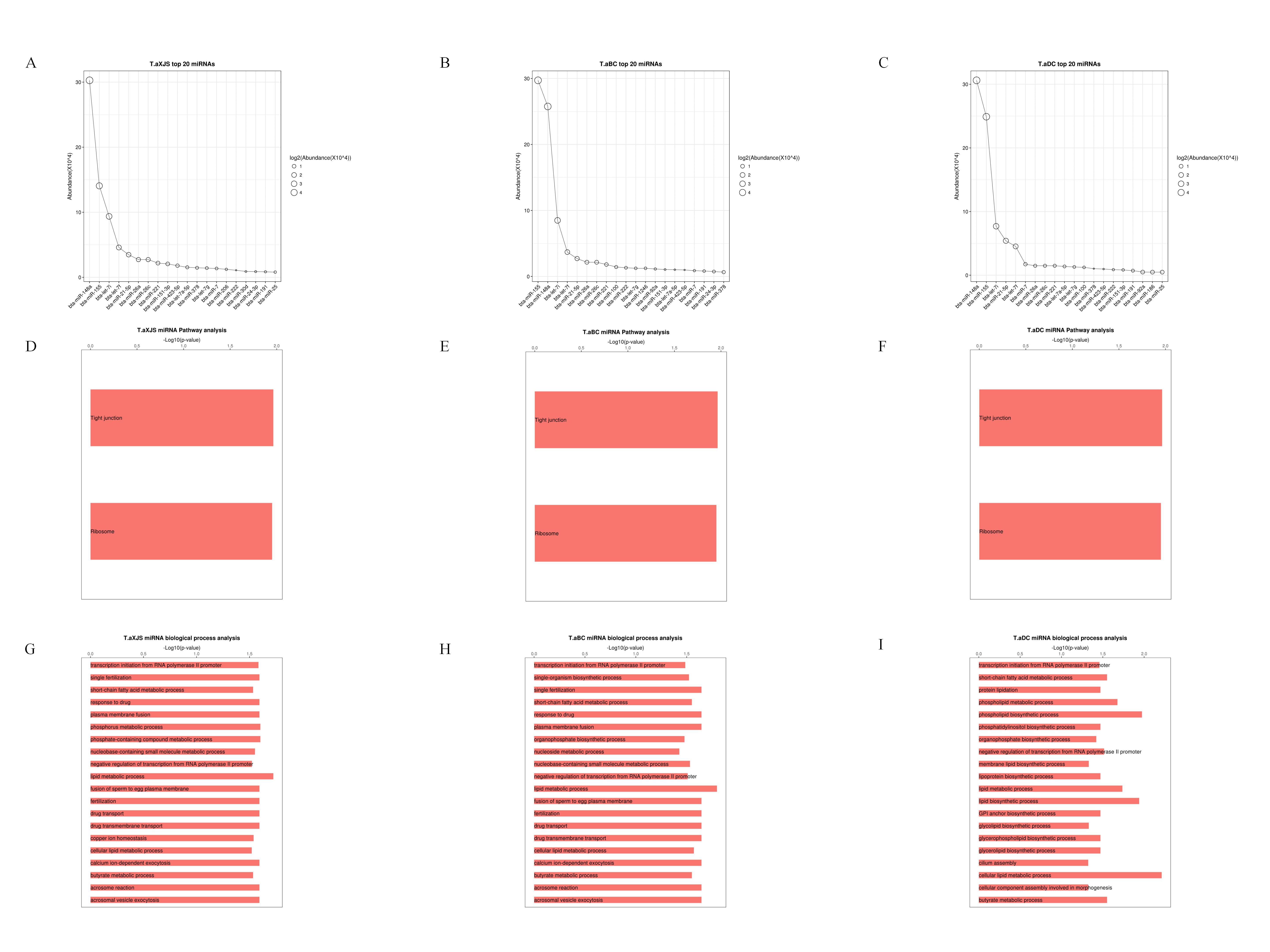

Supplement: Supplementary file 2 — Additional file 2. [file 13071_2025_6834_MOESM2_ESM.zip › Additional file 2-Fig. S6.jpg]

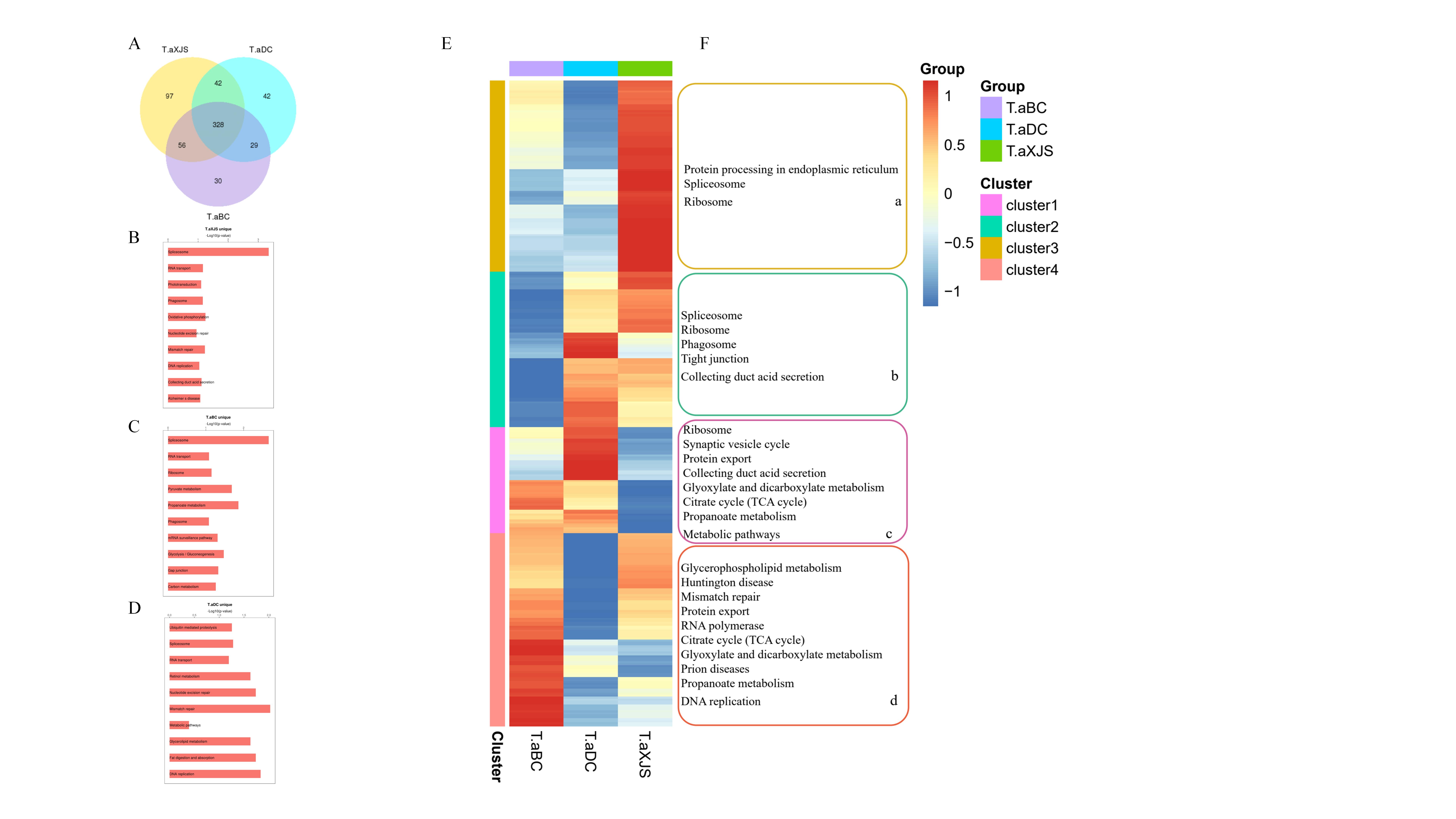

Supplement: Supplementary file 2 — Additional file 2. [file 13071_2025_6834_MOESM2_ESM.zip › Additional file 2-Fig. S7.jpg]

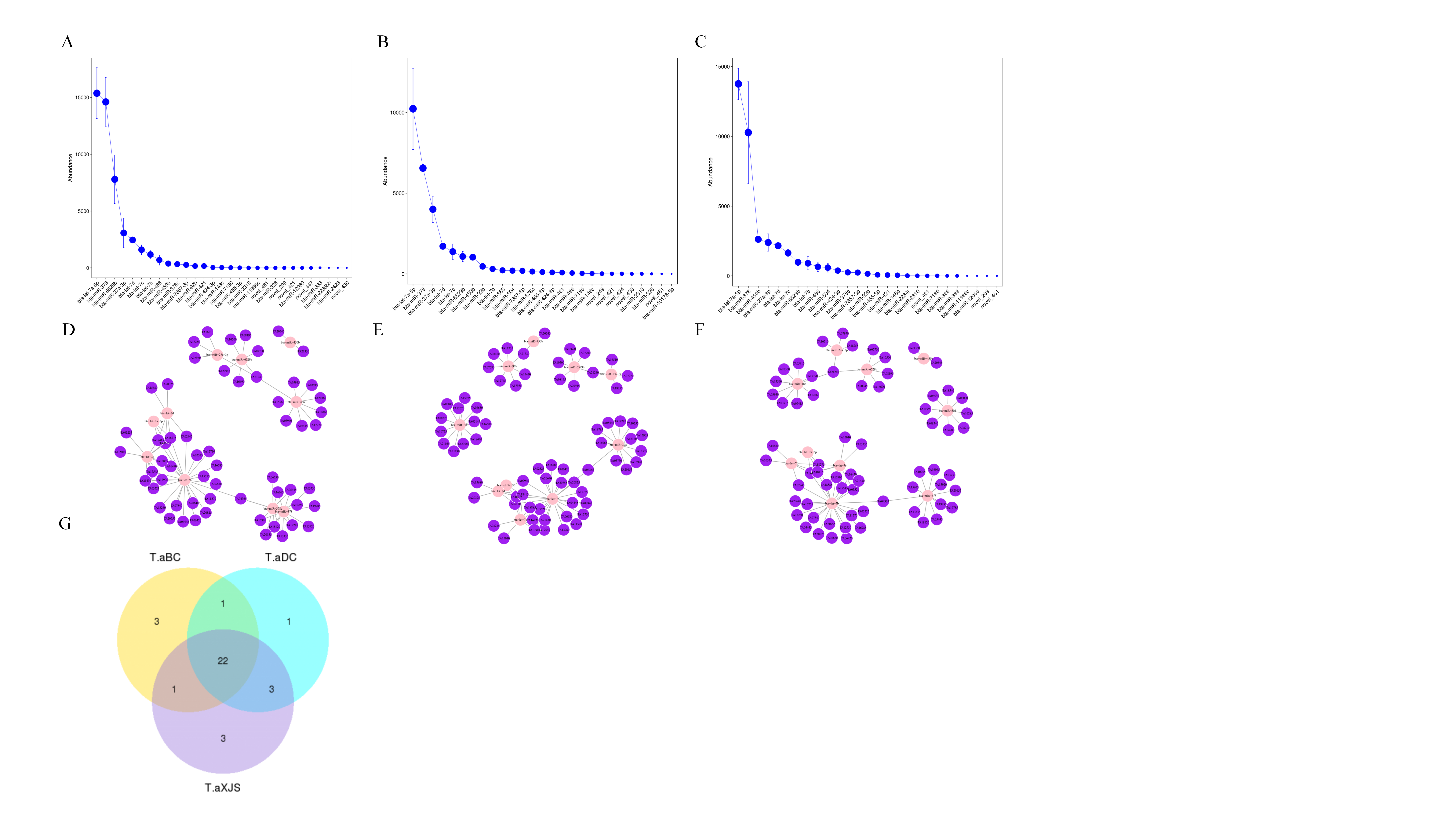

Supplement: Supplementary file 2 — Additional file 2. [file 13071_2025_6834_MOESM2_ESM.zip › Additional file 2-Fig. S8.png]

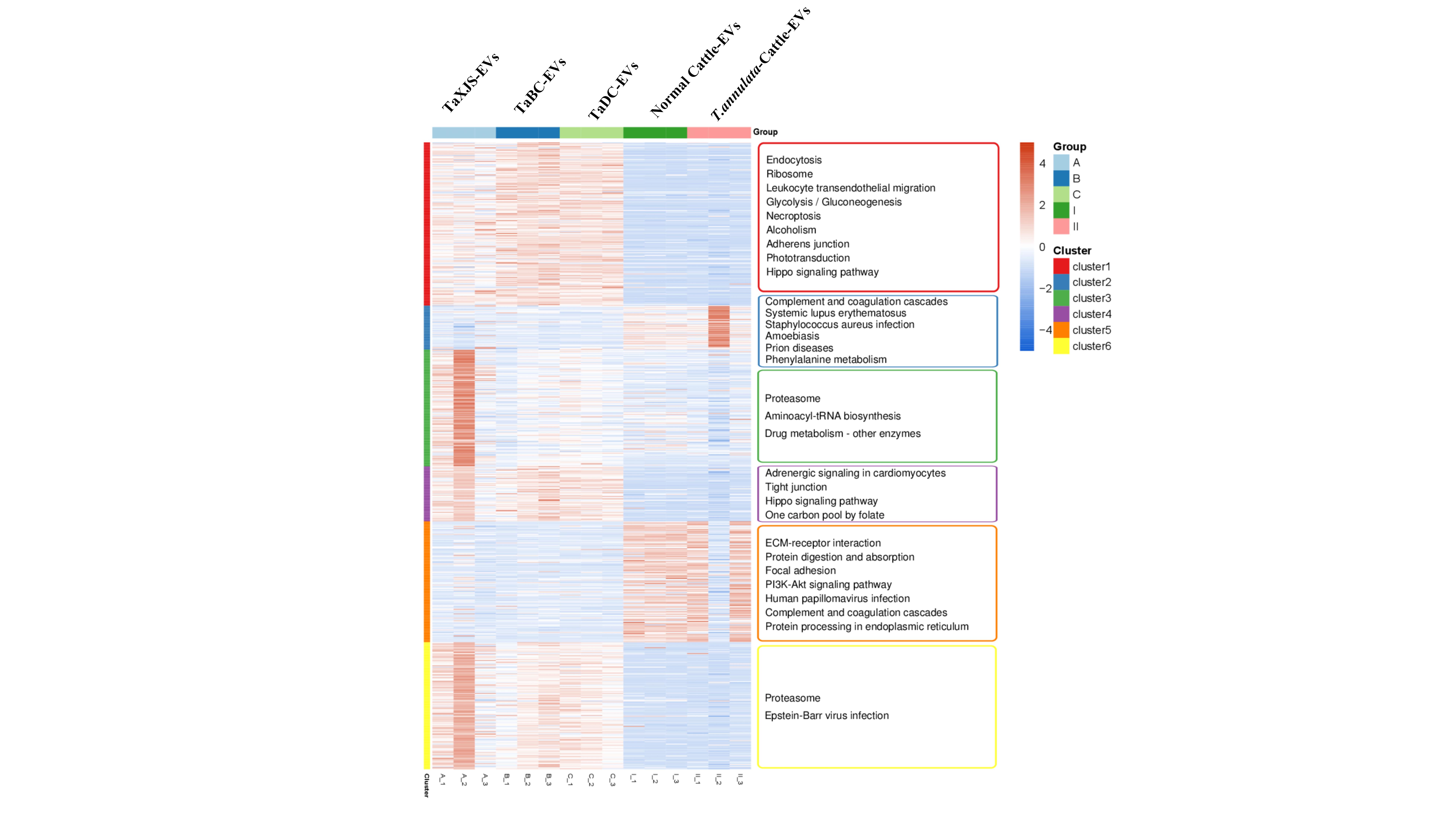

Supplement: Supplementary file 2 — Additional file 2. [file 13071_2025_6834_MOESM2_ESM.zip › Additional file 2-Fig. S9.jpg]

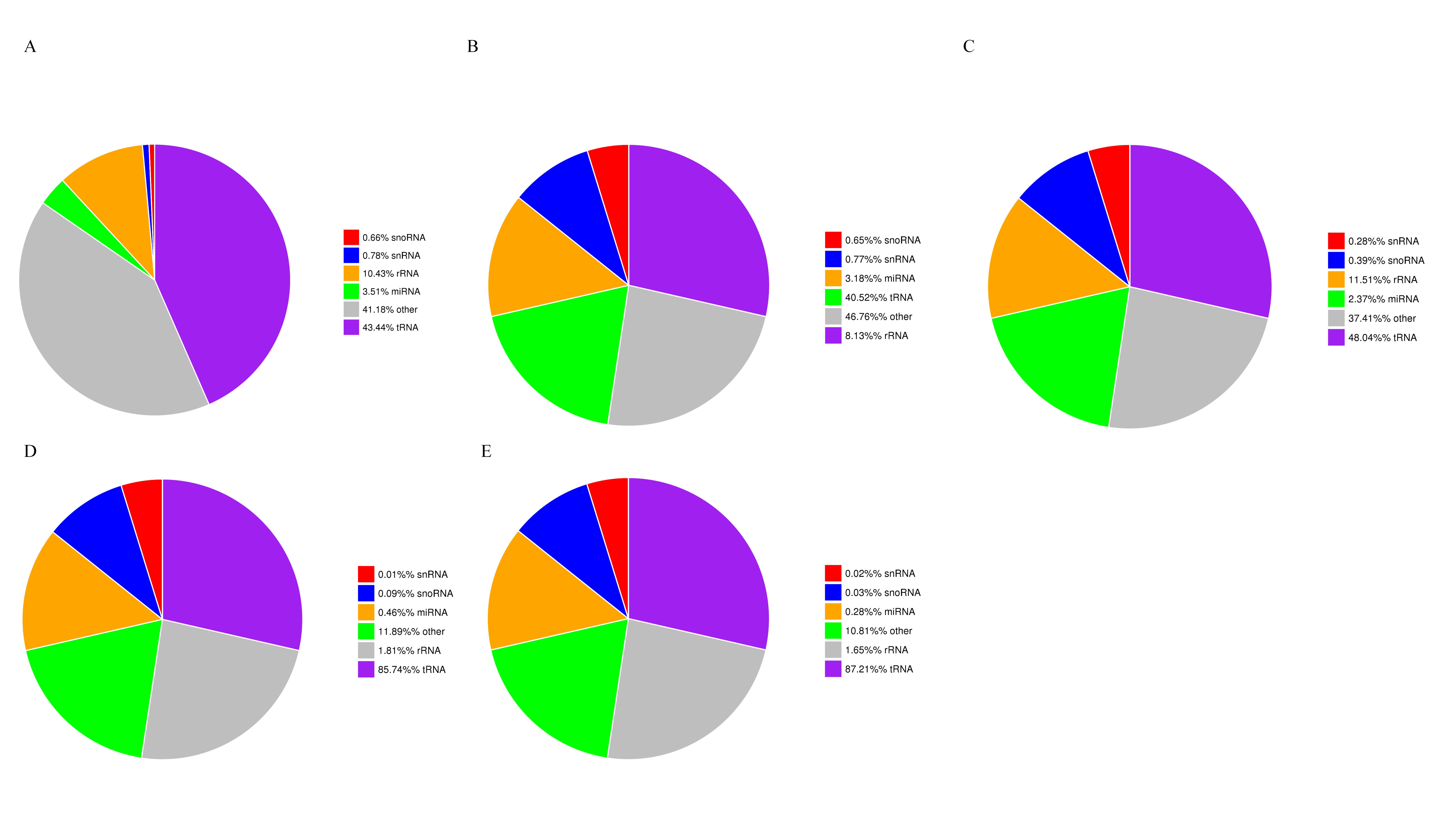

Supplement: Supplementary file 2 — Additional file 2. [file 13071_2025_6834_MOESM2_ESM.zip › Additional file 2-Fig. S10.jpg]

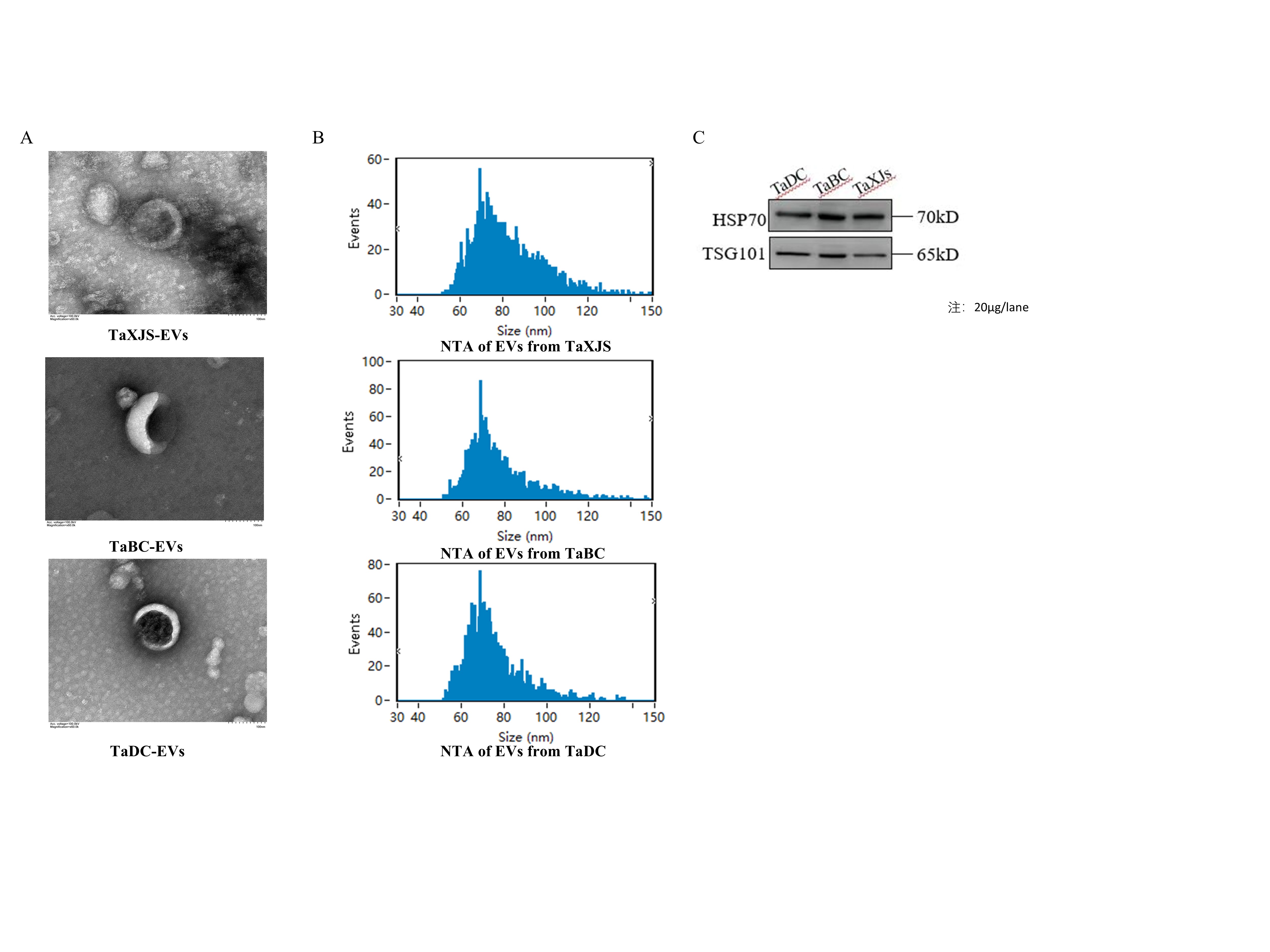

Supplement: Supplementary file 2 — Additional file 2. [file 13071_2025_6834_MOESM2_ESM.zip › Additional file 2-Fig. S1.jpg]

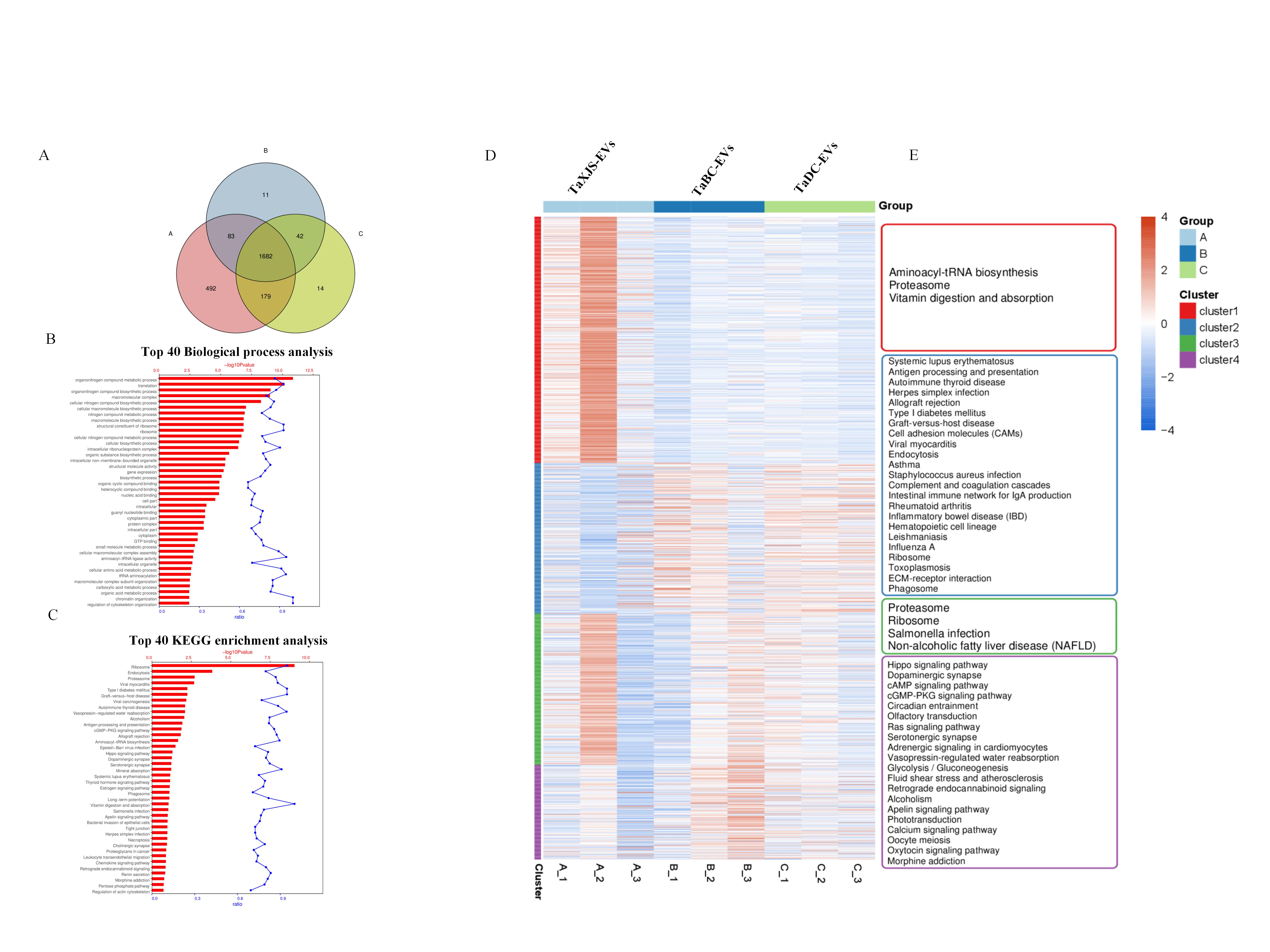

Supplement: Supplementary file 2 — Additional file 2. [file 13071_2025_6834_MOESM2_ESM.zip › Additional file 2-Fig. S2.jpg]

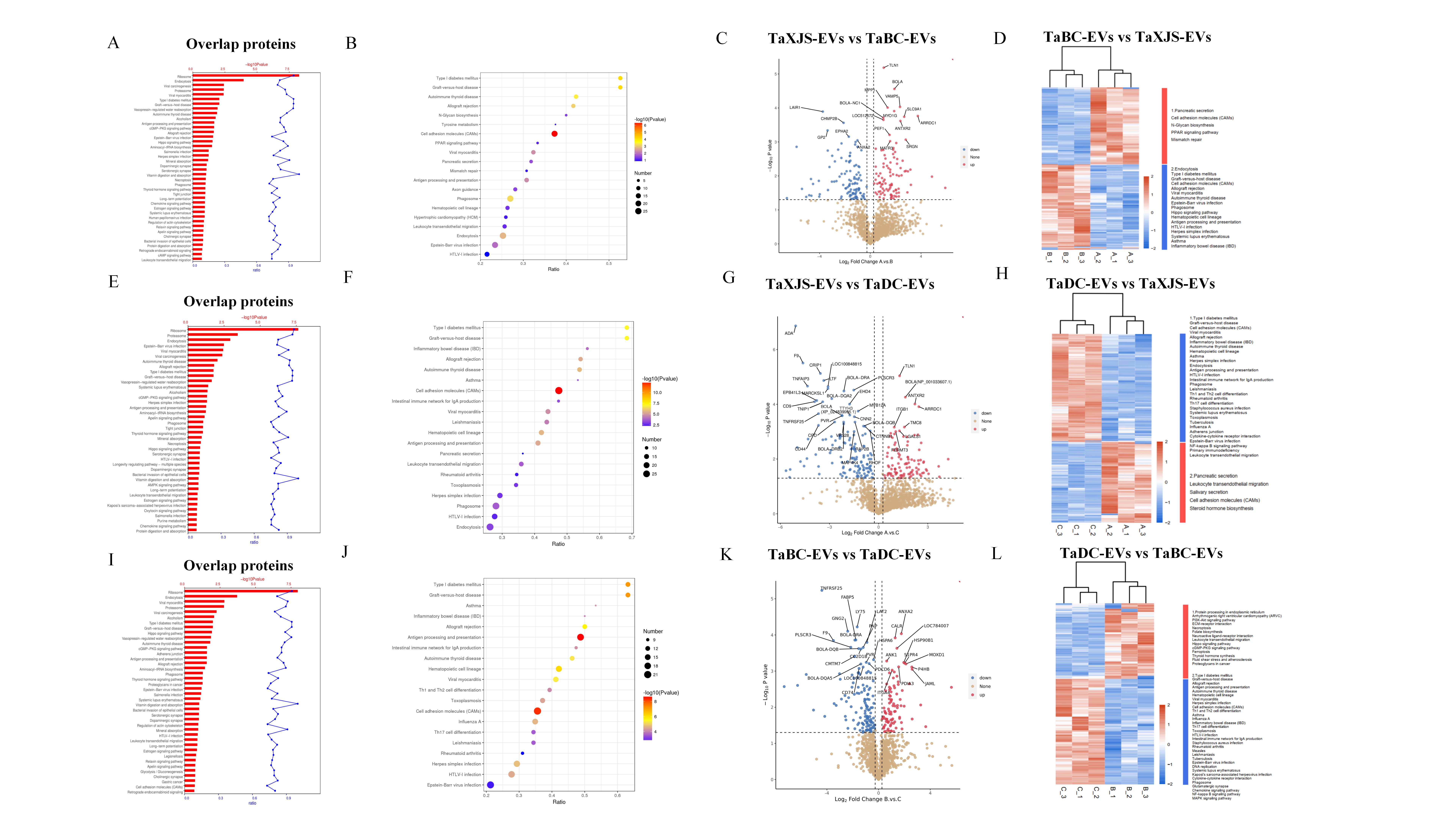

Supplement: Supplementary file 2 — Additional file 2. [file 13071_2025_6834_MOESM2_ESM.zip › Additional file 2-Fig. S3.jpg]

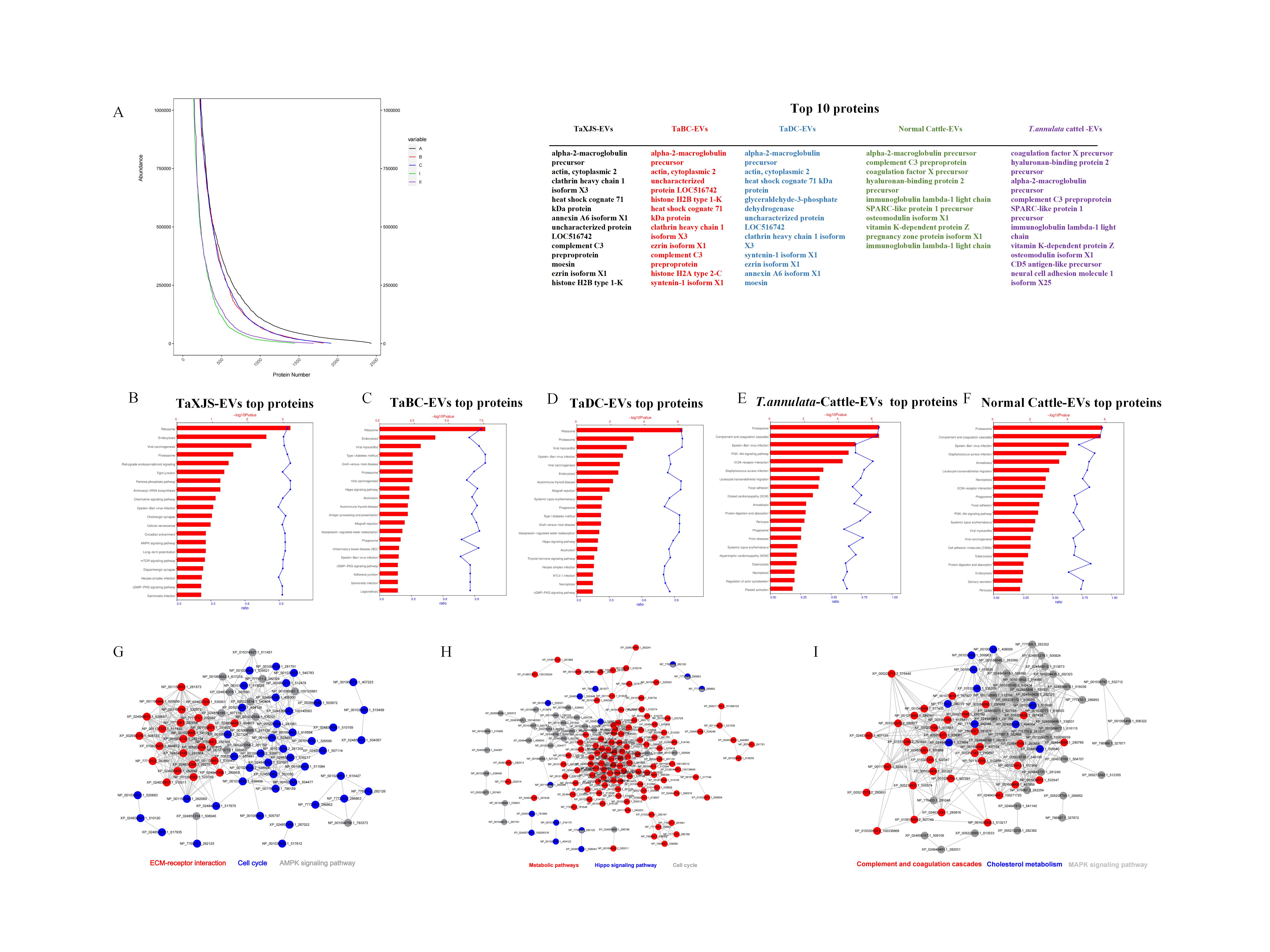

Supplement: Supplementary file 2 — Additional file 2. [file 13071_2025_6834_MOESM2_ESM.zip › Additional file 2-Fig. S4.jpg]

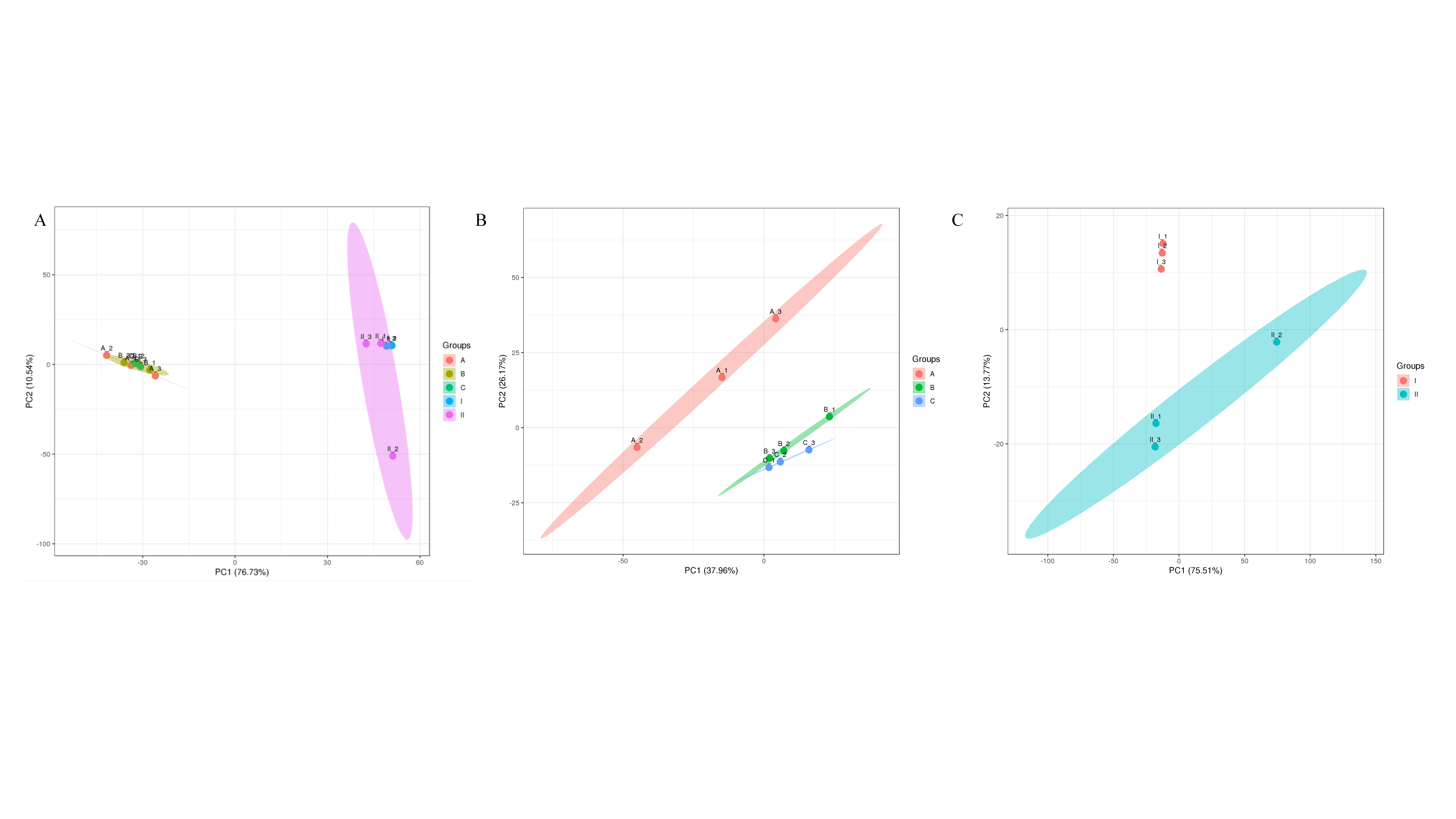

Supplement: Supplementary file 2 — Additional file 2. [file 13071_2025_6834_MOESM2_ESM.zip › Additional file 2-Fig. S5.png]
