# Supplementary material for: Systematic proteomic and small RNA profiling of extracellular vesicles from cattle infected with a naturally occurring buparvaquone-resistant strain of Theileria annulata and from uninfected controls
Source: Parasit Vectors. 2025 Jun 10;18:221. doi: 10.1186/s13071-025-06834-8 (PMC12153157; doi:10.1186/s13071-025-06834-8)
Supplement: Supplementary file 3 — Additional file 3. [file 13071_2025_6834_MOESM3_ESM.docx]

**Additional file 3:**

**Supplementary Table 1** All proteins of *Theileria annulata* identified in TaXJS-EVs, TaBC-EVs, TaDC-EVs by LC-MS/MS

| Uniprot ID | Description | Gene Names | TaXJS-EVs | TaBC-EVs | TaDC-EVs |
| --- | --- | --- | --- | --- | --- |
| XP_951813 | Actin 1 | TA15750 | Yes | Yes | Yes |
| XP_951820 | Uncharacterized protein | TA15710 | Yes | Yes | Yes |
| XP_951821 | CD8 T-cell antigen Ta9 | TA15705 | Yes | Yes | Yes |
| XP_951823 | Uncharacterized protein | TA15695 | Yes | Yes | Yes |
| XP_952563 | Heat shock 70 kDa protein | TA11610 | Yes | Yes | Yes |
| XP_952844 | U5 snRNP subunit, putative | TA09375 | Yes | Yes | Yes |
| XP_953048 | Glyceraldehyde-3-phosphate dehydrogenase | TA08145 | Yes | **None** | **None** |
| XP_953321 | Histone H4 | TA10535, TA10545 | Yes | Yes | None |
| XP_953473 | Oligomerisation domain containing protein | TA11275 | Yes | Yes | Yes |
| XP_953530 | Integral membrane protein | TA10420 | Yes | Yes | Yes |
| XP_953724 | 60S ribosomal protein L2/L8, putative | TA17025 | Yes | Yes | Yes |
| XP_953837 | Transitional endoplasmic reticulum ATPase (CDC48) | TA06500 | Yes | Yes | **None** |
| XP_954024 | Uncharacterized protein | TA06855 | Yes | Yes | Yes |
| XP_954051 | Elongation factor 1 alpha | TA06720 | Yes | Yes | Yes |
| XP_954237 | Uncharacterized protein | TA20390 | Yes | Yes | **None** |

*Note：* “Yes” indicates the presence of the specified protein in EVs of the corresponding cell line, while “None” denotes its absence.
