# Supplementary material for: Systematic proteomic and small RNA profiling of extracellular vesicles from cattle infected with a naturally occurring buparvaquone-resistant strain of Theileria annulata and from uninfected controls
Source: Parasit Vectors. 2025 Jun 10;18:221. doi: 10.1186/s13071-025-06834-8 (PMC12153157; doi:10.1186/s13071-025-06834-8)
Supplement: Supplementary file 4 — Additional file 4. [file 13071_2025_6834_MOESM4_ESM.docx]

**Additional file 4:**

**Supplementary Data**

**Identification of extracellular vesicles from TaXJS, TaBC and TaDC cells**

EVs were successfully isolated from the conditioned media of three *T. annulata*-infected bovine cell lines: TaXJS, TaBC and TaDC (Additional file 2: Fig. S1). These EVs were characterized based on their morphology, particle size, concentration and surface marker expression to confirm their identity and uniformity across the different cell lines. TEM revealed that the EVs displayed a typical cup-shaped morphology, with a size distribution from 40 nm to 150 nm, averaging around 100 nm, consistent with the expected characteristics of exosomes and other small EVs (Additional file 2: Fig. S1A). NTA was employed to quantify the concentration of EVs, revealing that TaXJS-EVs had the highest concentration at 1.76 × 10^11^ EVs/mL, followed by TaBC-EVs at 1.24 × 10^10^ EVs/mL, and TaDC-EVs at 1.83 × 10^10^ EVs/mL (Additional file 2: Fig. S1B). In addition to morphology, surface markers associated with exosomes were assessed using Western blot analysis. The positive markers TSG101 and HSP70 were detected in all three types of EVs, further confirming their identity as EVs (Additional file 2: Fig. S1C). Although the shape and size of EVs remained consistent across the cell types, significant differences were observed in their concentrations, suggesting that different cell lines may have distinct capacities for EV production.

**Pairwise comparison of extracellular vesicles proteomics**

Using LC-MS/MS, we identified 15 *T. annulata*-derived proteins within EVs isolated from the three infected cell lines (TaXJS-EVs, TaBC-EVs, and TaDC-EVs), as listed in Supplementary table 1 (Additional file 3-Table S1). Shared protein-coding genes across the different EV groups were analyzed using heatmaps, volcano plots, and Gene Set Enrichment Analysis (GSEA) to identify differentially expressed proteins (P < 0.05, |log2FC| ≥ 1) (Additional file 2: Fig. S2). Heatmap analysis revealed several common biological pathways across the TaXJS-EVs, TaBC-EVs and TaDC-EVs groups. In addition to shared pathways, several unique pathways were identified in each pairwise comparison. For instance, N-Glycan biosynthesis, PPAR signaling, mismatch repair, and autoimmune thyroid disease were specifically enriched in the comparison between TaXJS-EVs and TaBC-EVs (Additional file 2: Fig. S2D), whereas hematopoietic cell lineage, Shigellosis, intestinal immune network for IgA production, and steroid hormone biosynthesis were enriched in the comparison between TaXJS-EVs and TaDC-EVs (Additional file 2: Fig. S2H). Similarly, in the comparison between TaBC-EVs and TaDC-EVs (Additional file 2: Fig. S2L), pathways related to protein processing in the endoplasmic reticulum, PI3K-Akt signaling, ECM-receptor interaction, and neuroactive ligand-receptor interaction were notably enriched. Volcano plots (Additional file 2: Fig. S2CGK) illustrated significant protein expression differences between the various groups. When comparing TaXJS-EVs and TaBC-EVs, TaBC-EVs demonstrated upregulation of proteins associated with immune response regulation and cellular stress pathways, whereas TaXJS-EVs showed higher expression of proteins involved in metabolic processes and cell adhesion. Similarly, the comparison between TaXJS-EVs and TaDC-EVs indicated that TaDC-EVs had upregulated proteins linked to inflammation and cellular growth, while TaXJS-EVs were enriched for proteins involved in differentiation and structural integrity (Additional file 2: Fig. S2G). The comparison between TaBC-EVs and TaDC-EVs revealed that TaDC-EVs had elevated expression of proteins involved in cellular signaling and immune responses, whereas TaBC-EVs exhibited increased expression of proteins associated with metabolism and structural functions (Additional file 2: Fig. S2K). In serum samples, EVs from *T. annulata*-infected cattle showed enrichment in proteins related to nicotinate and nicotinamide metabolism, phagosome formation, and valine, leucine, and isoleucine biosynthesis.

**Bioinformatics of overlapping proteomes**

We described the expression abundance curve of the three types of EVs of cell lines and the EVs from sera of *T. annulata*-infected and uninfected cattle, then we identified the top ten gene symbols of loaded proteins in EVs (Fig. 3A). The proteomes of the five types of EVs were all involved in a complex signaling pathway regulatory network, and the top 20 pathways were then sorted based on the value of -log10 (P-value) (Additional file 2: Fig. S3B-F). All proteomes were enriched in the proteasome and Epstein-Barr virus infection. The protein-protein interaction (PPI) analysis revealed that the proteins enriched in the ECM-receptor interaction, cell cycle, and AMPK signaling pathways are densely interconnected, particularly in the ECM-receptor interaction. Similarly, in the metabolic and Hippo signaling pathways, there is a high degree of interaction among proteins, with metabolic pathways being the most enriched. These findings suggest that the proteins involved in ECM-receptor interaction and metabolic pathways play critical roles in maintaining cellular structure and metabolic regulation, while proteins in the cell cycle, Hippo signaling pathways, cholesterol metabolism and MAPK signaling pathway may regulate cell growth and proliferation (Additional file 2: Fig. S3G-I). To evaluate the difference between each two EVs proteomes, we constructed a Venn diagram to recognize the unique and overlapping protein-coding gene clusters (Additional file 2: Fig. S4). Bioinformatics analysis identified 1682 proteins shared among EVs from three distinct cell lines (Additional file 2: SFig.4A) that predominantly regulate key signaling pathways, including endocytosis, phagosome formation, and fatty acid metabolism. These shared proteins are primarily involved in a spectrum of extracellular activities and biological processes, such as organonitrogen compound metabolic process, threonine-type endopeptidase activity, extracellular space, organic substance catabolic process as detailed in Figure S4B. The heatmap analysis further revealed differences in the expression of shared proteins of TaXJS-EVs, TaBC-EVs and TaDC-EVs (Additional file 2: Fig. S4D, E). In Cluster 1, proteins involved in the Aminoacyl-tRNA biosynthesis, proteasome, Vitamin digestion and absorption signaling pathways were enriched in TaXJS-EVs (Additional file 2: Fig. S4D, E). Proteins in Cluster 2 mainly participated in the regulation of antigen processing and presentation, allograft rejection, cell adhesion, endocytosis, complement and coagulation cascades, the intestinal immune network for IgA production, hematopoietic cell lineage, ECM-receptor interaction, phagosome formation, and ribosome function. Additionally, these proteins were involved in pathways related to diseases such as leishmaniasis and toxoplasmosis in TaBC-EVs and TaDC-EVs. The TaXJS-EVs, TaBC-EVs and TaDC-EVs both contained Cluster 3 proteins, which regulated the proteasome, ribosome, *Salmonella* infection, and the non-alcoholic fatty liver disease. Proteins in Cluster 4, which were mainly enriched in TaBC-EVs and TaDC-EVs, participated in hippo signaling pathway, cAMP signaling pathway, cGMP-PKG signaling pathway, ras signaling pathway, glycolysis, apelin signaling pathway, calcium signaling pathway and oxytocin signaling pathway signaling.

**miRNA profiles of the three extracellular vesicles types**

The RNA content of EVs was analyzed using the Illumina SE50 platform. For an initial investigation of parasite-specific mRNAs within the EVs, total RNA from a highly purified EV sample was sequenced, and the data were curated using stringent thresholds. Principal component analysis (PCA) (Additional file 2: Fig. S5) was used to evaluate the reproducibility of miRNA expression levels across samples. Based on sequencing results, the top 20 miRNAs in each of the three EV types were identified (Additional file 2: Fig. S6A-C). Among these, bta-miR-148a, bta-miR-155, and bta-let-7i were the most abundant miRNAs across all three EV types. Beyond these dominant miRNAs, bta-miR-24-3p was present in both TaXJS-EVs and TaBC-EVs, bta-miR-25 in both TaXJS-EVs and TaDC-EVs, and bta-miR-100 and bta-miR-92a in both TaBC-EVs and TaDC-EVs. Notably, bta-miR-206 and bta-miR-30d were found exclusively in TaXJS-EVs, bta-miR-1246 only in TaBC-EVs, and bta-miR-186 only in TaDC-EVs. To further explore the biological functions of these miRNAs, we filtered the top miRNAs based on P < 0.05 and read counts > 1000. Target genes were then predicted, and these genes were subjected to Gene Ontology (GO) and Kyoto Encyclopedia of Genes and Genomes (KEGG) pathway enrichment analyses. The results indicated that miRNAs from TaXJS-EVs, TaBC-EVs, and TaDC-EVs significantly impact pathways related to tight junctions, ribosome function, transcription initiation from RNA polymerase II, short-chain fatty acid metabolism, lipid metabolism, and butyrate metabolism (Additional file 2: Fig. S6D-F). Common pathways affected by miRNAs from TaXJS-EVs and TaBC-EVs included single fertilization, drug response, plasma membrane fusion, and nucleobase-containing small molecule metabolism. TaBC-EVs and TaDC-EVs miRNAs commonly regulated the organophosphate biosynthetic process. In contrast, only TaXJS-EVs miRNAs significantly influenced pathways related to phosphorus metabolism, phosphate-containing compound metabolism, and copper ion homeostasis. TaBC-EVs miRNAs uniquely affected single-organism biosynthetic processes and nucleoside metabolism, while TaBC-EVs miRNAs also uniquely influenced pathways involved in protein lipidation, phospholipid metabolism, and biosynthesis, as well as glycerolipid biosynthesis, cilium assembly, and cellular component assembly involved in morphogenesis (Additional file 2: Fig. S6G-I).

**Unique and shared miRNAs of the three extracellular vesicle types**

We investigated the role of the unique miRNAs from the three EVs types in signal regulation. The Venn diagram revealed 97, 30, and 42 unique miRNAs in TaXJS-EVs, TaBC-EVs, and TaDC-EVs, respectively, and 328 shared miRNAs (Additional file 2: Fig. S7A). The regulatory network of miRNA-protein interactions was evaluated by targeting the unique miRNAs. In TaXJS-EVs, the 97 unique miRNAs were found to regulate phototransduction, oxidative phosphorylation, collecting duct acid secretion pathway, etc. (Additional file 2: Fig. S7B). For the 30 unique miRNAs in TaBC-EVs, KEGG analysis revealed multiple significant ontologies, including metabolism of pyruvate, mRNA surveillance pathway, propanoate metabolism, carbon metabolism, gap junction, etc. (Fig. 7C). In TaDC-EVs, the 42 unique miRNAs were found to participate in the regulation of ubiquitin mediated proteolysis, retinol metabolism, metabolic pathways, clycerolipid metabolism, fat digestion and absorption, etc. (Additional file 2: Fig. S7D). The miRNA components indirectly contribute greatly to multiple signaling pathways. Heatmaps were used to visualize the differentially expressed miRNAs across the three EV types. The heatmap illustrates the differential expression of miRNAs across TaXJS-EVs, TaBC-EVs, and TaDC-EVs (Additional file 2: Fig. S7E). Additionally, the number of differentially expressed miRNAs (P < 0.05 or P < 0.01) between each pair of EV types was evaluated. Distinct expression patterns are observed among the EV types, with protein processing in endoplasmic reticulum, spliceosome, ribosome miRNAs, phagosome, tight junction and collecting duct acid secretion highly expressed in TaXJS-EVs compared to TaBC-EVs and TaDC-EVs, highlighting potential functional differences. TaBC-EVs had significantly lower miRNA enrichment than other types of EVs in the regulation of phagosome, tight junction and collecting duct acid secretion. However, the degree of enrichment in DNA replication, propanoate metabolism, glyoxylate and dicarboxylate metabolism was significantly higher than that of other types of EVs. Pluripotency-related miRNAs (with abundance > 1000) in the three EVs types were identified and listed (Additional file 2: Fig. S8A-C). The regulatory networks of the top ten miRNAs and their associated pathways were also depicted (Additional file 2: Fig. S8D-F). Finally, Venn diagram analysis revealed 22 overlapping miRNAs, suggesting that these miRNAs could play a crucial role in regulating the pluripotency of cells infected with *T. annulata* (Additional file 2: Fig. S8G). To investigate the interaction between the unique miRNA clusters and canonical pathways, we performed a regulatory network analysis using the R package ggplot2. The regulatory network results revealed that the top read count miRNAs (P < 0.05, abundance > 1000) in the regulatory network were mapped to multiple canonical signaling pathways. Cytoscape was used to draw the network of unique miRNAs from the five EVs types and their regulated signaling pathways.
